# Standardized Whole Blood Assay and Bead-Based Cytokine Profiling Reveal Commonalities and Diversity of the Response to Bacteria and TLR Ligands in Cattle

**DOI:** 10.3389/fimmu.2022.871780

**Published:** 2022-05-23

**Authors:** Jérémy Lesueur, Sarah Walachowski, Sarah Barbey, Nathan Cebron, Rachel Lefebvre, Frédéric Launay, Didier Boichard, Pierre Germon, Fabien Corbiere, Gilles Foucras

**Affiliations:** ^1^ IHAP, Université de Toulouse, INRAE, ENVT, Toulouse, France; ^2^ Unité Expérimentale du Pin, INRAE, Borculo, Le Pin au Haras, France; ^3^ GABI, Université de Paris-Saclay, INRAE, AgroParisTech, Jouy-en-Josas, France; ^4^ ISP, Université de Tours, INRAE, Nouzilly, France

**Keywords:** cytokine, bead-based assay, cattle, inflammation, bacteria, MAMPs, whole blood stimulation

## Abstract

Recent developments in multiplex technologies enable the determination of a large nu\mber of soluble proteins such as cytokines in various biological samples. More than a one-by-one determination of the concentration of immune mediators, they permit the establishment of secretion profiles for a more accurate description of conditions related to infectious diseases or vaccination. Cytokine profiling has recently been made available for bovine species with the development of a Luminex^®^ technology-based 15-plex assay. Independently from the manufacturer, we evaluated the bovine cytokine/chemokine multiplex assay for limits of detection, recovery rate, and reproducibility. Furthermore, we assessed cytokine secretion in blood samples from 107 cows upon stimulation with heat-killed bacteria and TLR2/4 ligands compared to a null condition. Secretion patterns were analyzed either using the absolute concentration of cytokines or using their relative concentration with respect to the overall secretion level induced by each stimulus. Using Partial Least Square-Discriminant Analysis, we show that the 15-cytokine profile is different under *Escherichia coli*, *Staphylococcus aureus*, and *Streptococcus uberis* conditions, and that IFN-γ, IL-1β, and TNF-α contribute the most to differentiate these conditions. LPS and *E. coli* induced largely overlapping biological responses, but *S. aureus* and *S. uberis* were associated with distinct cytokine profiles than their respective TLR ligands. Finally, results based on adjusted or absolute cytokine levels yielded similar discriminative power, but led to different stimuli-related signatures.

## Introduction

The last decade has witnessed an increase in the popularity of sequencing-based technologies like genomics and transcriptomics (1, 2). They allow the description of the genome structure and its expression in multiple tissues, organisms, and biological conditions. However, without protein-level information, it may be difficult to interpret the results of receptor–ligand interactions. These interactions are particularly important for certain classes of immune proteins like cytokines, which are a group of small molecules mediating dialogs between cells involved in the functions of the immune system. The fine-tuned regulation of their expression often leads to short ranges of biological variations but strong physiological effects (3), so that their dosage requires highly sensitive and specific methods. These criteria are met by immune-based methods, such as enzyme-linked immunosorbent assay (ELISA), which is a common but time-consuming technique due to its low throughput. For high-scale detection and measurement of cytokines, several multiplexing techniques have been developed to determine concentrations in human and laboratory species, like the planar electro-chemiluminescence assay, known as the Mesoscale^®^ technology for its commercial version, or bead-based assay, like the Luminex^®^ technology (4).

Such assays are still scarce in most domestic species like ruminants, for investigating biological situations such as responses to inflammatory conditions, infectious diseases, or vaccination. Ruminants (cattle, goats, and sheep mainly) are of special interest because of the multiple services they provide to humans. However, they may also be sources of zoonotic diseases of public health concern (like tuberculosis, brucellosis, etc.). A central way of keeping good health in ruminant species may be a well-functioning immune system (5). Until recently, the study of their immunity was essentially conducted through serology in serum or milk. Antibody quantification is an easy way to assess exposure or immunity, and it is much more relevant to viruses due to the production of neutralizing antibodies that are the final effectors in clearing the infection. In contrast, for bacteria, antibodies may increase opsonophagocytosis, but neutrophils and macrophages are central to the bactericidal activity. Progress made in understanding the immune responses to pathogens has emphasized the importance of the cell-mediated response, besides the production of immunoglobulins. Activation of the cellular response, with both innate and adaptive arms acting in concert against a pathogen or after contact with microbial-associated molecular patterns (MAMPs), leads to the secretion of various kinds of cytokines. Profiling secretion enables the characterization of a specific response and is informative on the mechanisms mobilized by the organism to fight the pathogen-related disease. This method is already applied for the detection of exposure or chronic infection by some pathogens like *Mycobacterium* spp., *Mycobacterium tuberculosis*, and *M. bovis*, the causal agents of tuberculosis in humans and cattle, respectively. Screening for the presence of a mycobacterial infection relies on IFN-γ detection, a cytokine released during the cell-mediated response in infected organisms (6), but several other cytokines are also involved in the response to mycobacteria. These might be helpful to differentiate among the various disease or infection states (7), and thoroughly describe immunity to mycobacteria and its underlying mechanisms, as recently suggested (8). Several attempts were also made to assess bovine innate immunity using whole blood assays (9, 10).

Recent technologies such as the Luminex^®^, a multiplex bead-based immunoassay platform, meet the criteria for high throughput, sensitivity, and specificity. The technology uses magnetic or polystyrene microscopic beads to bind a specific target analyte. Beads are dyed with a given fluorophore, enabling the identification of the target. A second fluorophore enables us to quantify the amount of analyte bound to each bead. This method allows the quantification of up to 100 different analytes in a single sample. This high-throughput technology provides data at an unusual scale for the description of the cytokine response. Indeed, cytokine profiling is becoming more and more popular, and a recent description of highly perturbed cytokine secretion in patients with COVID-19 (11) or influenza (12) infection shows that the method of data analysis might be improved over the linear description of cytokine changes. Data clustering has been recently proposed to resolve this issue for mining cytokine profiles and ease their biological interpretation. Improved methodology, such as the Cytomod adjustment procedure (13), is required when a comparison of the cytokine profiles is made between several conditions, whether they are health-related conditions or laboratory-based assays.

In this study, we describe the assessment of the response to different stimulatory agents like bacteria and Toll-like receptor (TLR) ligands by measuring cytokine and chemokine secretion using a bead-based assay newly developed for the bovine species. We evaluated the assay performance independently of the manufacturer on samples prepared using a standardized whole blood assay on a large group of cattle. Here, we show that the bead-based multiplex assay combined with a multivariate analysis allows the discrimination of different kinds of stimuli used to induce cytokine secretion and describes a new methodology to exploit these data for an improved interpretation over existing methods.

## Materials and Methods

### Study Population

The study was conducted on 107 Holstein–Friesian cows from the Pin-au-Haras farm, an experimental dairy cow facility (doi: 10.15454/1.5483257052131956E12) of the French National Research Institute for Agriculture, Food and Environment (INRAE). The average age was 3.4 ± 1.0 years, and 44, 43, and 20 cows were respectively in their first, second and third or higher lactation, for an average parity number of 1.9 ± 0.9. Of the 107 cows, 28 were pregnant when sampled (23 ± 18 days). Animals were free from zoonotic infections like brucellosis and tuberculosis, and also free from bovine leukosis. Their health status has been carefully examined from two weeks before to two weeks after the blood drawing. Only minor health events (six mastitis, one cystitis, and one lameness case) were recorded in this time frame, and none yielded a divergent cytokine profile, so all data were kept for analysis. All cows were managed in a single herd and, hence, exposed to the same environmental factors.

### Sample Preparation and Stimulation

Blood sampling was performed at 101 ± 11 days post-calving to avoid the period of highest predisposition to infectious diseases due to immune depression in the month after calving, as previously reported (14). Blood was drawn at the jugular vein directly into 1.2 ml stimulation syringes coated with lithium heparin (S-Monovette^®^, Sarstedt Marnay, France), and containing 200 µl of 10× stimulating reagent in RPMI-1640 with 0.1% serum albumin and 10× penicillin/streptomycin. In this study, two types of stimulating agents were used: three heat-killed bacteria and three Toll-like receptors (TLR) ligands (**Table 1**). Heat-killed bacteria were *Escherichia coli* (HKEC), S*taphylococcus aureus* (HKSA), and S*treptococcus uberis* (HKSU). The TLR ligands were a synthetic diacylated lipoprotein named Fibroblast-Stimulating Lipopeptide 1 (FSL-1), lipopolysaccharide (LPS), and gardiquimod (GDQ) binding to TLR2/6, 4, and 7/8, respectively in cattle. All reagents were from InvivoGen (InvivoGen, Toulouse, France) except HKSU, which was a home-made preparation kindly provided by FB. Gilbert (ISP, INRAE, Nouzilly, France). Briefly, the HKSU 2211 strain was isolated from a bovine clinical mastitis and was characterized as belonging to the Multilocus Sequence Type (MLST) 6 (clonal complex 5, which is one of the most frequently isolated in Europe (15, 16)). After culture in Dulbecco’s modified Eagle medium and optical density determination, bacteria were heat-inactivated at 70°C for 50 min. Inactivation was verified by culture on blood agar Petri dishes.

**Table 1:** List of the stimuli used in the whole blood assay.

Stimulus	Abbreviation	Concentration	Source	Receptor
**Bacteria**				
HK *Escherichia coli O111:B4*	HKEC	10e7/ml	InvivoGen tlrl-hkeb2	complex
HK *Staphylococcus aureus*	HKSA	10e7/ml	InvivoGen tlrl-hksa	complex
HK *Streptococcus uberis*	HKSU	10e7/ml	HM	complex
**MAMPS**				
Fibroblast-Stimulating Lipopeptide 1	FSL-1	1 µg/ml	InvivoGen tlrl-fsl	TLR2/6
Imidazoquinoline	GDQ	3 µg/ml	InvivoGen tlrl-gdq-5	TLR7/8
Lipopolysaccharide	LPS	3 µg/ml	InvivoGen tlrl-3pelps	TLR4

HK, heat-killed; HM, home-made.

Stimulation began immediately upon collection and samples were incubated at 38.5°C (the rectal temperature of *Bos taurus* adults) for 24 h. At the end of the incubation period, tubes were spun down at 750 g for 10 min, and plasma samples were aliquoted into 96-well plates for immediate storage at −20°C.

### Ethics Statement

All procedures involving animals received approval from the Ethics Committee on Animal Experimentation (agreement No. 2016082518447444), with all applicable provisions established by the European directive 2010/63/UE. All methods were performed by approved staff members in accordance with the relevant standard operating procedures approved by the above-mentioned ethics committee. We handled all the animals used in this study in strict accordance with good clinical practices, and we made all efforts to minimize suffering.

### Cytokine and Chemokine Measurement

#### Sample Dilution

Plasma samples were defrosted at 37°C and carefully resuspended. Approximately 100 µl were transferred to 96-well conical bottom plates and centrifuged for 5 min at 450*g* to remove any debris that might be present. Then, 30 µl were transferred into new 96-well conical bottom plates and diluted to 1:10 with assaying buffer.

#### Bead-Based Multiplex Assay

Cytokine concentrations were determined with a custom 15-plex bovine cytokine assay developed by Merck-Millipore (SPRCUS617, Millliplex^®^ xMAP^®^, Merck-Millipore, France). The analytes consist of five cytokines involved in the innate response (IL-1α, IL-1β, IL-1RA, IL-6, and TNF-α), five of the adaptive response (IL-2, IL-4, IL-10, IFN-γ, and IL-17A), and five chemokines (CCL2 (MCP-1), CCL3 (MIP-1α), CCL4 (MIP-1β), CXCL8 (IL-8), and CXCL10 (IP-10)).

Assays were carried out on a set of eleven 96-well plates on six separate dates by a single operator by running a batch of two plates. Both plates of a batch contained the low and high concentration controls, but only one contained the seven-point standard. Controls and standards were assayed in duplicates, while the cow samples were assayed as single points.

Each plate was first washed with 200 µl of wash buffer under agitation for 10 min at room temperature. For the standards and controls, 25 µl of bead solution (vortexed beforehand) was placed into each well, and 25 µl of standard or control solution and 25 µl of matrix serum (total volume of 75 µl) were added. For samples to be determined, wells were filled with 25 µl of bead solution, 25 µl of assay buffer, and 25 µl of diluted plasma as indicated above. Plates were covered with plastic film and aluminum foil before incubation under agitation (500–700 rpm) overnight at 4°C. At the end of the incubation, the plates were washed 3 times with a wash buffer. The beads were retained by placing the plate on a magnetic support 1 min before the supernatant harvest and during the entire wash procedure. Then, 25 µl of detection solution was added to each well. The plates were covered with plastic film and incubated under agitation (500–700 rpm) for 1 h at room temperature. To reveal the secondary antibody, 25 µl of streptavidin–phycoerythrin were added and incubated for 30 min at room temperature. Plates were washed 3 times as previously described. Finally, the beads of each well were resuspended in 150 µl of sheath fluid, and the plates were covered with plastic film and incubated for 5 min at room temperature.

#### Data Acquisition

To obtain the median fluorescence intensities (MFI), fluorescence intensities were recorded on a MAGPIX^®^ system (Luminex^®^) equipped with a CCD camera and processed by the xPONENT^®^ software (version 4.2.1324.0, Luminex Corp, Austin TX USA).

### Statistical Analysis

#### Method Validation

The pre-study validation was performed as described by Findlay et al. (17). Following the recommendations of Breen et al. (18), analyses were carried out on raw MFI, i.e., without considering the background values. A four- or five-parameter logistic function was used to fit the dose–response curves to the standard samples:


$$f\left(x,b,c,d,e,f\right)=c+\frac{d-c}{{\left(1+10^{b\left(x-e\right)}\right)}^{f}}$$

with *x* the concentration, *b* the opposite of the slope of the function at the inflection point, *c* the lower asymptote, *d* the upper asymptote, *e* the concentration producing a halfway response between *c* and *d*, and *f* the shape parameter (fixed at 1 in the case of a four-parameter logistic function).

The choice between a four- or five-parameter regression curve was based on the Akaike Information Criterion (AIC). Parameter estimation was performed using the Levenberg–Marquardt algorithm (19), weighted by the mean of MFI values. This weighting assumes a response–error relationship, i.e., the function linking the mean to the MFI value standard deviation, following a power-of-the-mean (POM) function (17):


$$\sigma \left(y\right)=aE{\left(y\right)}^{b}$$

with *y* the analyte concentration, *σ* the standard deviation of the response, *a* the proportionality constant, *E* the mean response, and *b* the shape parameter.

This assumption was checked for each analyte with linear regression. The goodness of fit of the model was assessed from the standardized residuals values and distributions.

For each cytokine, the recovery rate of the control samples was computed according to the following formula:


$$\tau =\frac{C_{est}}{C_{exp}}\times 100$$

with *C_est_* the concentration estimated from the dose–response curves and *C_exp_* the expected concentration of the given control sample. The recovery rate was considered correct if it was comprised between 75 and 125%⁠ (17).

The precision was estimated by computing coefficients of variation (CV). Assuming that the MFI values associated with a given standard assay follow a log-normal distribution, the CV was approximated with the following formula (20):


$$CV=\sqrt{e^{{\left(\sigma \times ln\left(10\right)\right)}^{2}-1}}$$

with *σ* the standard deviation of log-transformed MFI. The values of *σ* were computed by combining a bootstrap and a delta method to ensure a better approximation. The CV was computed for each cytokine and the six plates containing standards.

The “S”-shape of the logistic function decreases precision as concentrations reach the asymptotes. Hence, it is necessary to determine the limits of quantification within which measurements are reliable. These limits correspond to concentrations whose coefficient of variation does not exceed 25% (17), and were determined for each cytokine on the six plates containing standards.

#### Stimuli Discrimination

To investigate the ability of the multiplex cytokine assay to discriminate between different stimuli applied to blood samples, several approaches were investigated, based on estimated concentration values and raw or adjusted to the mean MFI values.

Concentration values were computed with *drLumi* (21, 22). Values outside the quantification range were assigned the upper (lower) limit of quantification when above (below) the range. MFI and concentration values were log10-transformed to handle the heteroscedasticity inherent to immunoassay data.

Adjustment for individual mean cytokine levels was performed after Cohen et al. (13) and adapted to several stimuli conditions. This adjustment makes it possible to obtain the stimulus-related relative cytokine level by considering the average cytokine level secreted by each individual for a given stimulus. More specifically, this adjustment follows two steps:

1 *Standardization of logged values*. This step was carried out on each cytokine separately, including data from all stimuli. The mean of the standardized values was then computed for each individual and each stimulus, which gives, ∀*i*∈〚1,*n*_*i*_〛,∀*j*∈〚1,*n*_*j*_〛 and ∀*k*∈〚1,*n*_*k*_〛, with *n_i_* the number of individuals, *n_j_* the number of stimuli, and *n_k_* the number of cytokines:


$$m_{i,j}=\frac{1}{n_{k}}\sum_{k=1}^{n_{k}}x_{i,j,k}$$

*m_i_*,*_j_* being the mean of the standardized values for individual *i* and stimulus *j*, and *x_i_*,*_j_*,*_k_* the standardized value for individual *i*, stimulus *j*, and cytokine *k.*

2 *Linear regression and residuals retrieving.* Finally, a linear regression was performed for each cytokine using the non-standardized logged values *y_i_*,*_j_*,*_k_* and the mean standardized cytokine values *m_i_*,*_j_* as the dependent and independent variables, respectively:


$$y_{i,j,k}=m_{i,j}\beta_{k}+\epsilon_{i,j,k}$$

where *β_k_* is a scalar and *ϵ_i_*,*_j_*,*_k_* the residual for individual *i*, stimulus *j*, and cytokine *k*.

The final adjusted values were the residuals *ϵ_i_*,*_j_*,*_k_*.

Pairwise comparisons of raw and adjusted concentrations or MFI values were first performed, using the Wilcoxon signed-rank test for paired data, with Holm–Bonferroni correction for multiple comparisons.

Further, multilevel Partial Least Squares Discriminant Analyses (PLS-DA) (23) were carried out, identifying the most discriminating factors for, on one hand, bacterial stimuli and, on the other hand, TLR ligands.

The multilevel approach was applied to consider the non-independent sampling structure of the data by focusing on the within-individual variations induced by stimulations and removing the between-individual variations (23, 24). The number of components chosen was the minimum that allowed a misclassification rate of less than 10%, computed from 10-fold cross validation run 100 times. The involvement of each cytokine in the characterization of the different stimulation conditions in relation to each other was evaluated by analyzing their contribution to the definition of the PLS-DA components and by computing their variable importance in projection (VIP) score (12).

Finally, multilevel sparse PLS-DA (sPLS-DA) (25) analyses were conducted to define the minimal set of cytokines allowing the best discrimination between the different stimulation conditions.

#### Statistical Software

All statistical analyses were performed on R (version 3.6.3) (26). Assay validation was performed using the *drLumi* package (version 0.1.2) (21, 22), an R package dedicated to multiplex immunoassay data analysis, was used to performed assay validation. The *mixOmics* package (version 6.10.8) was used to perform PLS-DA and sPLS-DA analyses (27).

## Results

### Characteristics and Validation of the Multiplex Assay for Bovine Cytokines/Chemokines

**Figure 1:**
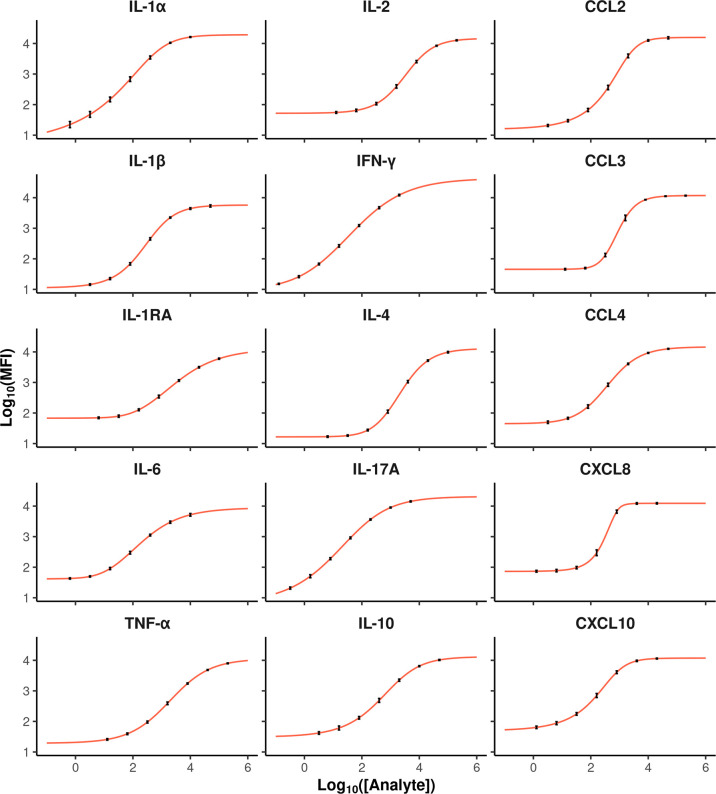
Average dose–response curves for each cytokine (red curves). The x and y-axis represent analyte concentration and the Log10 MFI value, respectively. Each dot corresponds to the average response of a given standard, and the error bars show the variances calculated on values from 6 plates for each cytokine.

Plasma supernatants from whole blood stimulations were assayed using the multiplex bovine cytokine/chemokine xMAP^®^ assay in a batch of 11 plates. Each run contained a 7-point standard curve and high/low concentration controls that were used to evaluate the performance of the assay. For the external standards, the goodness of fit was correct for all dose–response curves according to the standardized residuals values and their distributions (**Supplementary Figures S1**, **S2**). Besides, weighing the Levenberg–Marquardt algorithm was justified since the means and variances of MFI are well linked by the POM function (17) (**Supplementary Figure S3**). The characteristics of the logistic functions (number of parameters and parameter estimates) are provided in **Supplementary Table 1**. The curve slopes were gradual except for CXCL8 and CCL3, which showed a steep slope (**Figure 1**). The six fitted dose–response curves of each cytokine barely differed from one plate to another.

**Figure 2:**
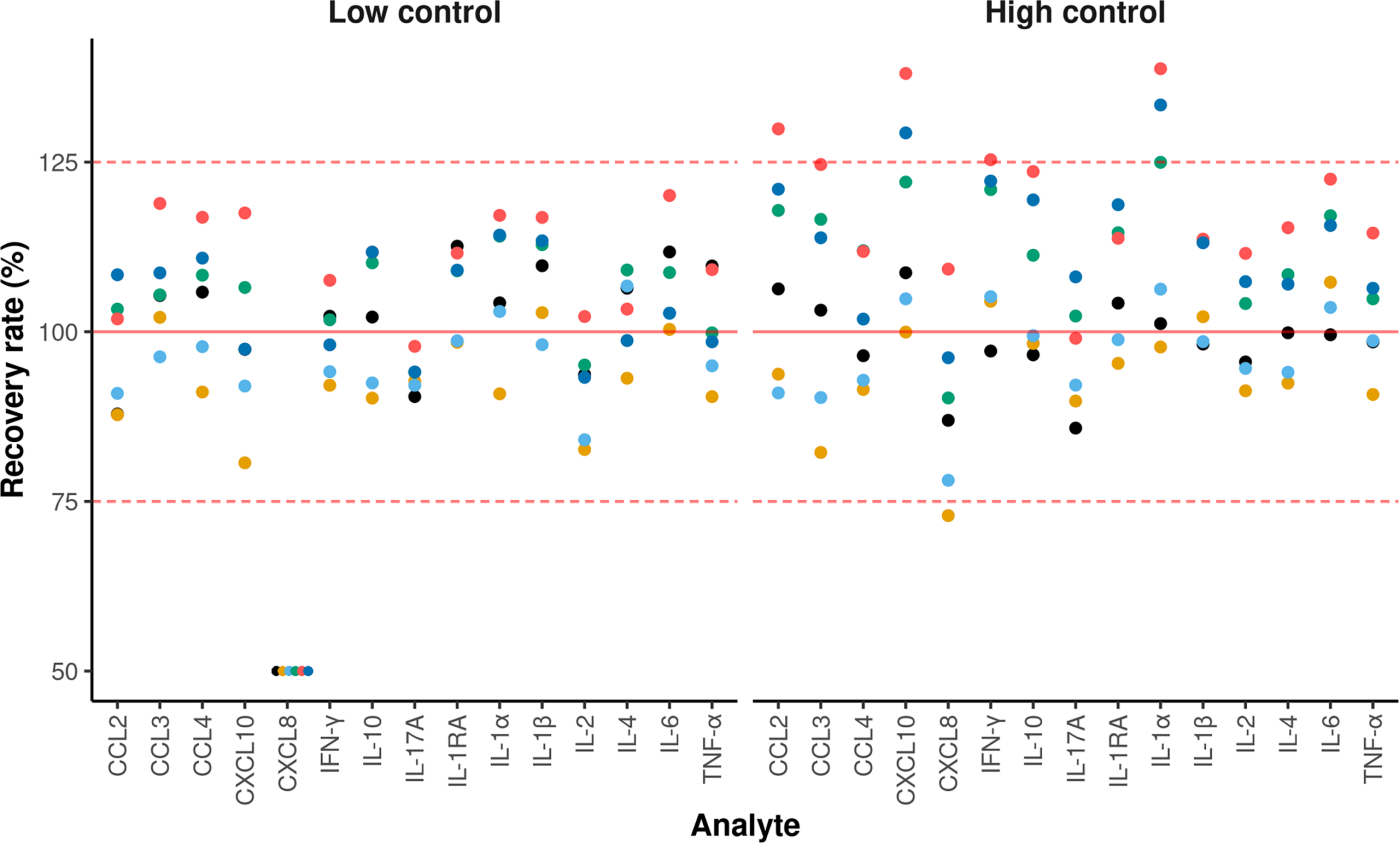
Recovery rates for each cytokine. The x and y-axis represent the analytes and the recovery rate, respectively. Each dot represents the recovery rate of a given plate. The solid red line indicates a 100% recovery rate. A recovery rate is considered correct when the value is between 75 and 125% (dashed red lines). For display purposes, all the samples having a recovery rate of less than 50% were set to 50%. Each color corresponds to one of the six plates containing control samples.

**Figure 3:**
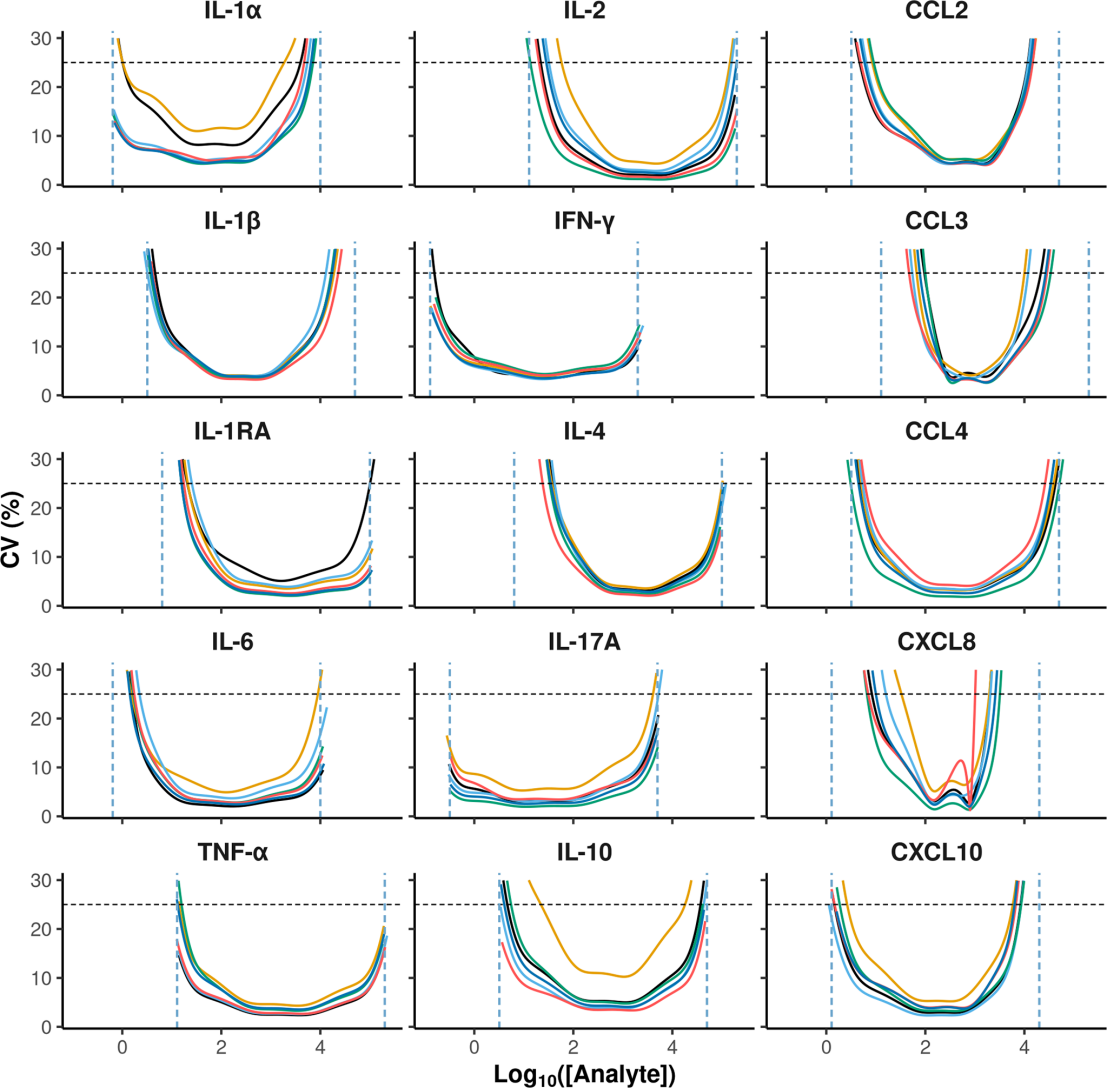
Coefficient of variation of MFI estimates as a function of cytokine concentrations. The x and y-axis represent analyte concentrations in Log10 and the coefficient of variation in percentage, respectively. The values of 6 separate plates are represented in different colors: horizontal black dashed lines indicate the 25% threshold that was used to define the reliable lower and upper quantification limits represented by the vertical blue dashed lines.

The accuracy criterion was met for all cytokines with a few exceptions (**Figure 2**). Indeed, it is generally admitted that a correct recovery rate should be comprised between 75 and 125% for an immunoassay (5). This was the case for all cytokines in the low concentration control except for CXCL8, for which the recovery rate was below 50%. For the high concentration control, the recovery rate was above 125% for some cytokines on two plates (CCL2, CXCL10, IFN-γ and IL-1α, CXCL10 and IL-1α), and below 75% on one plate (CXCL8). According to the CV profiles (**Figure 3**), precision was similar and correct across the plates, except for two plates. At the cytokine level, the precision was correct over a wide interval, except for CCL3 and CXCL8. Hence, for a given cytokine, the limits of quantification were quite constant across the plates (**Table 2**). For CCL3 and CXCL8, the ranges of quantification were tighter and their limits more widely distributed.

**Table 2:** Limits of quantification (in pg/ml).

Analyte	LLOQ min	LLOQ max	ULOQ min	ULOQ max
**CCL2**	5	9	11,768	14,639
**CCL3**	47	100	10,015	33,893
**CCL4**	3	6	26,168	50,000
**CXCL10**	1	3	5,842	8,612
**CXCL8**	7	33	1,012	3,190
**IFN-γ**	0	0	2,000	2,000
**IL-10**	3	22	18,022	46,218
**IL-17A**	0	0	4,068	5,000
**IL-1RA**	16	25	100,000	100,000
**IL-1α**	1	1	1,862	7,106
**IL-1β**	3	5	12,945	22,908
**IL-2**	14	58	145,399	200,000
**IL-4**	24	42	92,296	100,000
**IL-6**	1	2	8,910	10,000
**TNF-α**	13	16	179,671	200,000

The minimal lower (LLOQ min) and maximal lower (LLOQ max) limit of quantification, and the minimal upper (ULOQ min) and maximal upper (ULOQ max) limit of quantification across the plates are shown.

When the samples prepared with the whole blood assay were assessed, some MFI values were outside the quantification ranges in the set of 107 cows, except for TNF-α and IL-4 at the selected dilution (**Supplementary Table 2**). However, even if an MFI value beyond this range cannot be reliably converted into the concentration unit, it is still meaningful. Indeed, the edges of their distributions are not truncated, except for CXCL8, which means the beads are not saturated and these values reflect the amount of cytokines in a semi-quantitative way (15).

### Whole Blood Stimulation With Various Ligands Using An All-In-One Method

To evaluate the cytokine response profile of the group of more than one hundred cows, we adapted the conditions of a previously described blood assay developed for human studies (28). The material design enables sterile blood collection in the field and assays away from laboratory conditions. As mastitis is the major disease of dairy cattle that develops after infection of the mammary gland by three main types of bacteria like *E. coli*, *S. aureus*, and *S. uberis*, we investigated the blood cytokine response after stimulation by each of these three bacteria. Additionally, the responses to two main ligands for TLR2/6 and TLR4, which are known receptors for these bacteria, and also the response to a ligand for TLR7/8, a receptor which binds to single-stranded RNA, were assessed in parallel. The best concentrations for leucocyte activation were adapted from published studies and our own previous experience (**Table 1**). A null condition was also included with very low background measurements (**Supplementary Table 3** **)**. Results obtained using either MFI values or estimated concentrations were in strong agreement, and only those obtained for MFI values are detailed.

### Increased Production of Cytokines is Detected in All Stimulation Conditions Over the Null Control

**Figure 4:**
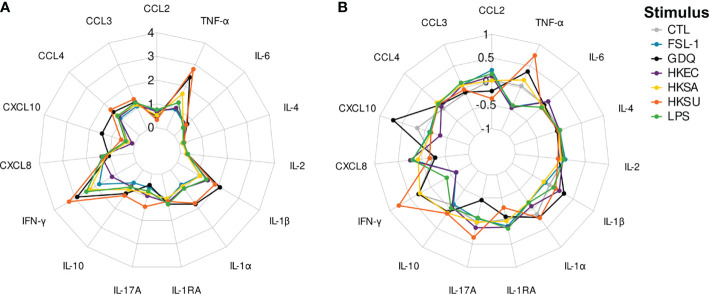
Radarplots of cytokine concentration values obtained for each stimulus. **(A)** represents the log10-transformed fold-changes induced by a stimulus over the null condition. **(B)** represents the adjusted concentration values. CTL, FSL-1, GDQ, HKEC, HKSA, HKSU, and LPS stand for control, fibroblast-stimulating lipopeptide 1, gardiquimod, heat-killed *Escherichia coli*, heat-killed *Staphylococcus aureus*, heat-killed *Streptococcus uberis*, and lipopolysaccharide stimuli, respectively.

In the null condition, several chemokines like CXCL8 and CCL3 were already at higher concentrations than generally assayed in bovine plasma samples without incubation (data not shown). In all stimulation conditions, stimulated blood samples consistently yielded increased levels of each cytokine compared to their paired unstimulated null controls (**Supplementary Figure 4**). Among the different conditions evaluated in parallel, GDQ and HKSU were associated with the greatest overall increase in cytokine levels, whereas FSL-1, LPS, HKEC, and HKSA induced secretion changes of lower and similar magnitude (**Figure 4A**). Notably, blood stimulation induced biological responses for IFN-γ and TNF-α spanning close to or greater than 500-fold compared to the null condition. Variations in CCL2, IL-2, IL-4, and IL-6 levels occurred in lower (<10 fold) but still significant magnitudes compared with null conditions (**Figure 4A** and **Supplementary Figure 4B**). For the conditions using bacteria, cytokines that are generally associated with inflammatory conditions like IL-1α and β, IL-6, and TNF-α were detected at increased concentrations, as expected. Adaptive cytokines like IFN-γ and IL-17A were also detected, although low expression levels were measured for IL-17A in some conditions. Chemokines were also strongly up, like CXCL8, CCL3, and CCL4. Overall, the results show that the standardized blood assay is efficient at producing blood cell activation and cytokine secretion.

### Adjusted Cytokine Levels Have Discriminative Capacity to Differentiate Among Bacterial Conditions

The null condition was further excluded from the following analysis to focus on the response to stimuli, and we looked for the best discriminative approach to compare the conditions. Several approaches were compared, based on raw or adjusted cytokine levels. This adjustment was performed following Cohen et al. (13): cytokine levels were regressed against the average cytokine level induced by each stimulus, leading to an individual stimulus-dependent cytokine profile depicting the relative abundance of each cytokine (**Figure 4B**). These profiles partially differed from those based on raw (unadjusted) values, highlighting the cytokines with the greatest positive or negative relative variation for each stimulation condition. For instance, while IL1-RA did not significantly differ between HKSU and HKSA stimulation conditions (p=0.427) when based on raw MFI values, its relative abundance was much higher under the HKSA stimulation condition (p<10^−15^). Sometimes, the direction of pairwise differences was even reversed, as observed for CCL3 or CCL4 under HKSU, HKSA, and HKEC stimulation conditions, with significantly higher raw MFI values but lower relative abundances when blood was stimulated with HKSU.

### Multilevel PLS-DA Improves Discrimination Among Bacteria Stimulation and Enables Identification of the Most Contributing Cytokines

**Figure 5:**
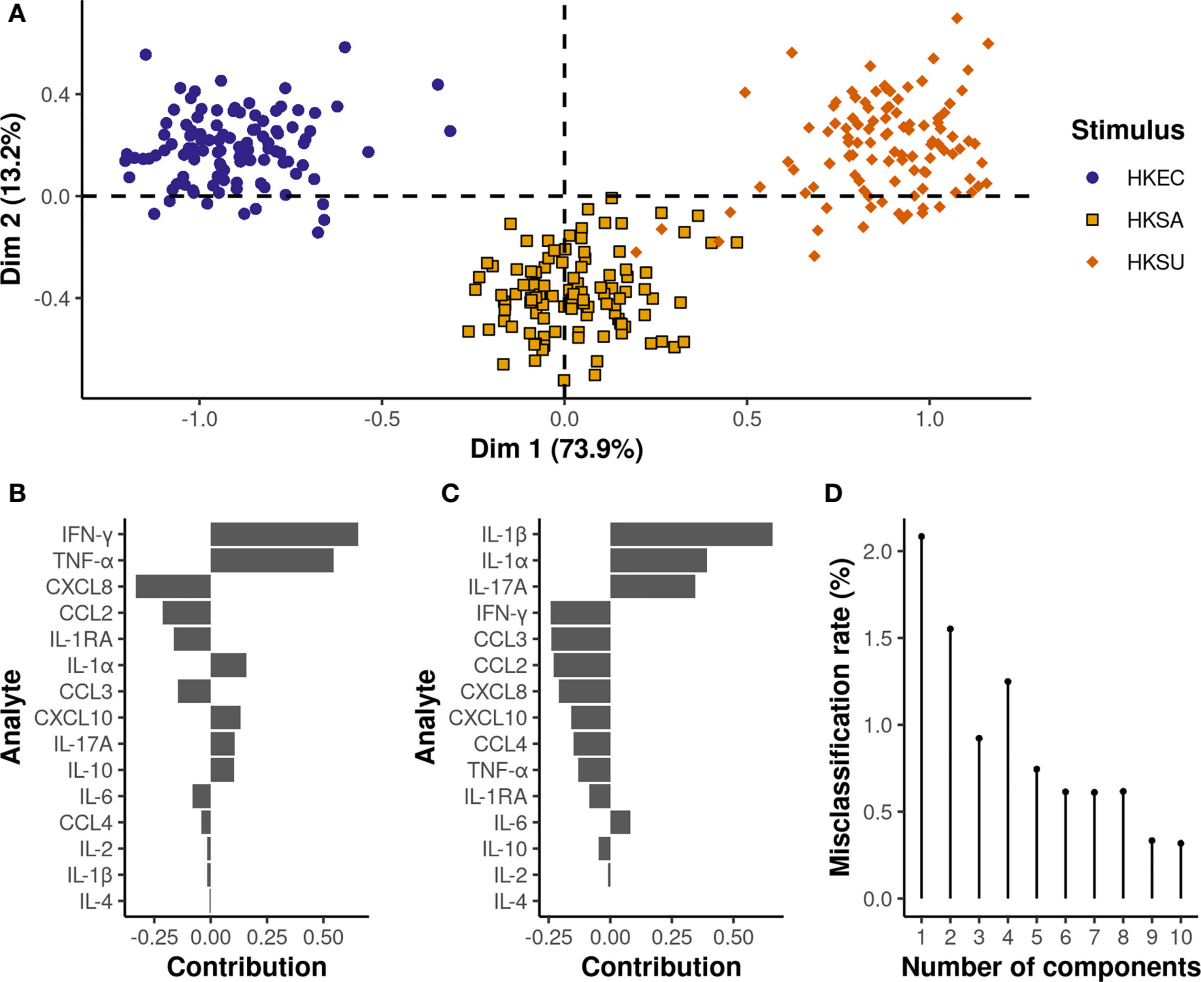
Multilevel PLS-DA on bacterial stimuli adjusted MFI values reveals specific expression patterns depending on the bacteria species. On the plot of the individuals **(A)**, each dot represents an individual cow, and the corresponding stimulus is defined by its color. The x and y-axis correspond to the first and second PLS-DA components, respectively. The proportion of variance explained by each principal component is written in brackets. The contribution of the cytokines to the first **(B)** and second **(C)** principal components is represented by a horizontal barplot. The two first principal components are sufficient to achieve an almost perfect misclassification rate **(D)**.

According to the multilevel PLS-DA applied to adjusted MFI values, the multiplex cytokine immunoassay allowed for clear discrimination of the three bacterial stimuli, with a misclassification rate of 1.5% on the two first components (**Figure 5**). Cytokines with high variable importance in projection scores (VIP>1) were the most significant and contributed most to the first two PLS-DA components (**Supplementary Figure 6A** **)**. IFN-γ, TNF-α, IL-1α, IL-1β, and CXCL8 were considered the most discriminant cytokines, with higher IFN-γ, TNF-α, IL-1α, and lower CXLC8 relative levels under HKSU stimulation conditions than under HKSA and HKEC conditions, respectively. Compared to both HKEC and HKSU, HKSA was further characterized by lower IL-1β and IL-1α relative levels. IL-17A (VIP=0.94 on the first two components) also significantly contributes to the discrimination of the three bacterial stimuli, with relative levels being the highest under HKSU conditions and the lowest under HKSA conditions. Pairwise comparisons (**Supplementary Figure 5** **)** revealed other features that also contributed to the discrimination of the three bacterial stimuli, but to a lesser extent (VIP<0.9) (**Supplementary Figure 6A**). For instance, significantly higher relative levels of CXCL10 were found for HKSA and HKSU conditions compared to HKEC, while no difference was found between HKSA and HKSU, explaining the low contribution of this cytokine to the first two PLS-DA components. In the same way, both HKEC and HKSA conditions yielded significantly higher CCL3 relative levels compared to HKSU, while no difference was evidenced between HKEC and HKSA.

Results from the sPLS-DA analysis revealed that quite good discrimination between the three bacterial stimulation conditions could be achieved on the basis of only three cytokines, i.e., IFN-γ, IL-1β, and TNF-α, with a misclassification rate of 5.6% based on the first two components, and 1.6% using the first three (**Supplementary Figure 7**).

### TLR2 and TLR4 Ligands Share Commonalities Between Them and With Bacteria

**Figure 6:**
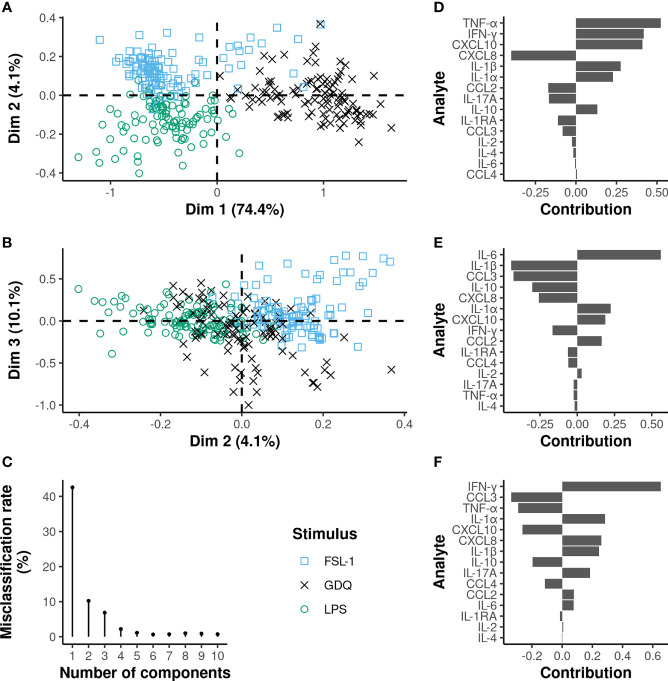
Multilevel PLS-DA on TLR ligands adjusted MFI values identifies commonalities between TLR2/6 and TLR4 engagements. On the plots of the individuals **(A, B)**, each dot represents an individual cow and the corresponding stimulus is defined by the different colors and shapes. The x and y-axis correspond to the PLS-DA components. The proportion of variance explained by each principal component is written in brackets. The contribution of the cytokines to the first **(D)**, second **(E)** and third **(F)** principal components are represented by a horizontal barplot. The three first principal components are sufficient to reach a misclassification rate below 10% **(C)**.

The same analysis was also applied to TLR ligand stimulations, leading to a misclassification rate of 10.2% on the two first components of the PLS-DA analysis and 6.8% on the first three (**Figure 6**). The first component, explaining almost 75% of the total variance, allowed a clear distinction of the GDQ stimulation condition, whereas the second component mainly contributed to differentiating FLS-1 and LPS conditions. The third component refines the discrimination by compensating for the small overlap between GDQ and FSL-1 on the first component. Almost all cytokine relative levels significantly differed between GDQ and both FSL-1 and LPS conditions (**Supplementary Figure 5**), with higher TNF-α, IFN-γ, IL-1β, and CXCL10, and lower CXCL8 and CCL-3 relative levels contributing most to the characterization of the GDQ condition (VIP>1 on the first 3 components) (**Supplementary Figure 6C**). FSL-1 and LPS conditions could be differentiated mainly on the basis of higher IL-6 relative levels and, conversely, lower levels of IL-1β for LPS compared to FSL-1. Again, the sPLS-DA analysis showed that the relative levels of TNF-α and IL-6 were enough to differentiate the three TLR ligands with a misclassification rate of 8.4% on the first 2 components (**Supplementary Figure 8**).

**Figure 7:**
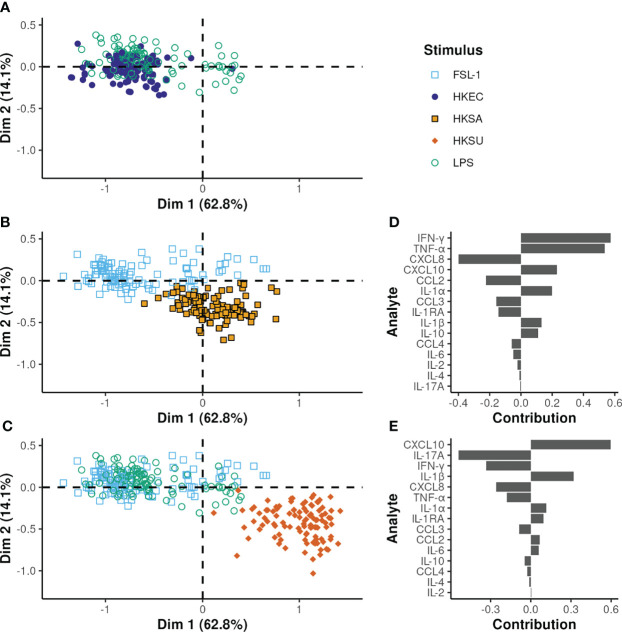
Multilevel PLS-DA on all bacteria and TLR ligands except GDQ. **(A–C)** output of the same PLS-DA but displaying only one bacterial stimulus and the TLR ligands known to activate the same TLR. The x and y-axes correspond to the first and second PLS-DA components, respectively. The proportion of variance explained by each principal component is given in brackets. **(D, E)** the variable contributions to the first and second components, respectively.

As expected, the cellular responses to HKEC and LPS conditions were overlapping **(** **Figure 7A**). Notably, HKSA and HKSU showed different response profiles from TLR ligands known to activate the same TLR **(** **Figures 7B, C**).

### Raw MFI Values Have Similar Discriminative Capacity Using PLS-DA

When applied to raw (non-adjusted) MFI values, the PLS-DA analyses led to mostly the same conclusions, with the identification of TNF-α, IFN-γ, and IL-1β contributing most to the discrimination of the three bacterial stimuli (VIP>1 on the first two components, and a misclassification rate of 0.7% on the first two components) (**Supplementary Figures 9** and **6B**). Small differences were, however, evidenced regarding the involvement of CXCL8, with a VIP score of 0.63 and 1.1 on the first two components when non-adjusted and adjusted MFI values were analyzed, respectively. Other differences in the stimulus-related signatures were also demonstrated for IL-6, CCL3, CCL4, IL-1RA, and CXCL10 but contributed to a lesser extent in the discrimination of the different bacterial stimuli. Similarly, IFN-γ, CXCL8, TNF-α, IL-1 α, IL-1β, CXCL10, and IL-6 were identified to contribute most to the discrimination of TLR ligands (VIP>1 on the first three components, misclassification rate of 3.7% on the first three components) when unadjusted MFI values were analyzed (**Supplementary Figures 10** and **6D**). CCL3 contributed less significantly to TLR ligand discrimination when unadjusted MFI values were analyzed (VIP score 0.8 vs 1.12 on the first 3 components), while the opposite situation was demonstrated for IL-1α (VIP score 1.26 vs 0.9).

## Discussion

We evaluated a new multiplex assay based on the Luminex^®^ technology developed for cattle and used it to assess the cytokine profile in blood samples stimulated with bacteria of three different genera. These bacteria are highly prevalent in the environment of cattle and are commonly involved in the development of mastitis, a mammary gland inflammation that is the most frequent disease in dairy herds. We also collected profiles using two MAMPs, LPS and FSL-1, which are ligands of TLR4 and TLR2/6, respectively, and GDQ, an endosomal TLR7/8 ligand that engages different signaling pathways. The profiles are in agreement with the previously reported data, with some additional observations (28). Production of innate cytokines like IL-1β, IL-6, and IL-8 was previously reported in response to three TLR ligands (LPS, Pam3CSK4, and R848 or resiquimod) using a whole blood assay in cattle (9). Several observations can be made from the comparison of our results with those of previous studies. First, the background is noticeably lower for IL-1β and IL-6 in our study compared with the previously reported data (9, 10), indicating that further manipulation after blood drawing is associated with cell activation and non-specific cytokine production. Our all-in-one approach enables us to reduce the non-specific secretion of cytokines for a better definition of responsiveness. Second, the inter-individual variation reported there was much higher than what we observed, although a higher production of cytokines in response to LPS was detected (10). This may be due to activation by ligands other than LPS itself, as high TLR2 activity is detected in some commercial sources of LPS (29). To increase specificity, we used ultrapure LPS as a TLR4 ligand. FSL-1, which is shown to be more stable than Pam3CSK4, was used for specific TLR2 activation. The TruCulture*^®^* method, which is the most advanced development for testing the blood response in humans, has also been used in cattle for the same purpose (10). The concentrations used in previous studies and ours are closely related and different from those used to stimulate human or murine cells, indicating that the stimulation conditions need to be adapted to the bovine species. Ready-to-go commercial reagents for human studies may not be optimal to evaluate the bovine innate response as it was previously done in calves (10), except if the concentrations are the same as for the presently used bacteria. Furthermore, the array of pathogens that infect *Bos taurus* is essentially different from that of human beings, and this is another reason why reagents developed for human studies may be irrelevant to assessing bovine responsiveness due to a different co-evolution with their host.

In contrast, to most of the studies that describe the expression at the mRNA levels (30–32), we measured secreted cytokine and chemokine proteins. Indeed, most reports describe the expression of cytokine genes in various kinds of cell types upon stimulation with bacteria or their MAMPs. The results of these assays have different meanings and discrepancies between mRNA and protein expression are often reported (33–35); it is worth remembering that only proteins support biological functions, making changes in their quantities more insightful information. Thus, we have established a system protocol that is an improvement over existing methods used to establish cytokine expression profiles. The method can be implemented almost anywhere where an outlet is available to connect the heat block for incubation, and it has a low background and high specificity and accuracy in quantification.

First, the use of an all-in-one method of blood collection and stimulation considerably reduces the cytokine background, as previously indicated in human studies (28, 36). Limiting the manipulation of the blood samples to set up the stimulation reduces the technical variability and improves reproducibility, improving the reliability of the measure. This is even more marked when the expression profile of PBMCs prepared over a Ficoll cushion is compared with that obtained from the whole blood (37–39). Second, the use of a Luminex^®^ technology-based assay enables the quantification of analytes with a low variance compared to ELISA. From a statistical viewpoint, MFI values provide more insightful information than concentrations do (18), although the latter are more accessible to the biologist for interpretation. Moreover, the automatic calibration step gives a reproducible signal output (40), enabling the comparison of MFI values obtained from different experiments across different MAGPIX^®^ devices. Comparisons of values obtained through other devices require conversion to concentrations. Third, a new method of data exploitation using available modules enables statistical analysis, and the reduction of information to facilitate interpretation of medium and high throughput results in a completely free-of-charge R environment (26).

The bacteria used for blood stimulation are extracellular pathogens commonly involved in the development of bovine mastitis. Infections by extracellular bacteria are usually detected through the release of MAMPS and the ensuing stimulation of TLR and dectin receptors (41, 42). In contrast, heat-killed bacteria do not have access to the cytosol and may not be able to engage endogenous TLRs. Indeed, part of the immune activation is due to the interaction of some of the TLR agonists like those assayed in our study with their specific receptors. Evaluated in parallel on the same cell source, we showed that heat-killed *E. coli* and LPS, the main ligand for TLR4, induced very similar profiles, indicating that a large part of the response to *E. coli* is actually mediated by TLR4 engagement. In contrast, TLR2 is a receptor for lipoproteins usually found in the cell walls of Gram-positive bacteria like *S. aureus*, which is a prototypic bacteria signaling through this receptor. HKSA and FSL-1 have essentially divergent profiles, indicating that other signals from the killed bacteria are detected by leukocytes and induce a different profile of the cytokine response. The same observation prevails for *S. uberis*, another Gram-positive coccus involved in mastitis development. One possibility is the preferential activation of lymphocyte subsets by the two later bacteria, and lymphocyte-derived cytokines may contribute to feed-forward loops and specific microbial-associated signatures that are different from TLR ligand-only signatures, as previously suggested (43).

Most studies based on the analysis of cytokine profiles either characterize differences in immune response between groups of patients with different severity scores or progression stages (44–47), or try to discriminate between healthy and diseased individuals (44–47). Much attention has also been paid to the characterization of pathogen-specific cytokine profiles in both human and veterinary medicine (12, 48, 49). Differences in pathogen-related cytokine profiles can be revealed by simple pairwise comparisons of absolute cytokine concentration across different diseases or stimulation conditions, but multidimensional data analysis often provides a more in-depth view (12, 28, 50–52). Among various statistical approaches, non-supervised Principal Component Analysis (PCA) has been the most extensively applied, while supervised approaches, namely, PLS-DA, Random Forest, and Support Vector Machine data analysis, have been less widely explored as a means to interpret cytokine profiling (45, 53–56).

As far as we are aware, the characterization of stimulus-specific cellular immune responses based on parallel whole blood stimulation and multiplexed bead-based cytokine profiling on the same individuals has seldom been performed (28). Indeed, the diversity of immune responses in relation to different stimuli is often based on samples from independent individuals. When several stimulation conditions are applied in parallel to blood samples from the same individual or patient, the outcome of interest is most often independent of the stimulation conditions themselves (i.e., different severity scores, progression stages) (44–47). Analysis of multidimensional data derived from multiple stimulus conditions deserves appropriate statistical analysis. First, because cytokine levels are measured in blood samples from the same individual under different stimulation conditions, intra-individual correlation induced by repeated measures must be considered. This was performed by applying a multilevel PLS-DA approach, which separates between-subject from within-subject variations, before applying the multivariate approaches to the within-subject variation matrix, which is assumed to represent the treatment (i.e., stimulation condition) effect. Although other data integration methods may be applied (57), the multilevel approach has been successfully used in a range of situations with repeated multidimensional biological data (55, 58). In our study, disregarding the intra-individual correlation while leading to the definition of the same stimulus-related cytokine profiles was associated with a lower discriminative power, with a misclassification rate of 7.2% on the first two components for bacterial stimuli and 21.2% on the first three components for TLR ligand stimuli, when based on the analysis of adjusted MFI values (data not shown).

More importantly, since we are interested in defining stimulus-specific signatures, the relative amounts of each cytokine to each other may appear more informative than their absolute amounts. Indeed, in our experiment, HKSU and GDQ stimuli were associated with higher overall cytokine amounts than HKEC or HKSA stimuli. Disregarding the effect of each type of stimulation on the overall level of cytokines leads to distinguishing stimuli on the basis of the absolute number of cytokines they induce, not on the relative importance of cytokines to each other. While the analysis of adjusted or non-adjusted (absolute) responses led to the identification of the same major cytokines in the definition of stimulus-related signatures and similar discriminative power, different interpretations of these profiles emerged. Similar to Duffy et al. (28) in healthy human donors, we found that HKEC and LPS induced a strong pyrogenic cytokine response in cattle with increased TNF-α and IL-1β concentrations, which were induced by 10 to 1,000-fold compared to the null condition. However, in our experiment, the most potent stimuli, marked by the highest levels of these cytokines, were HKSU and GDQ, and IL-6 concentrations were only moderately increased by 3 to 5 times as compared to the null condition.

The definition of stimuli-related signatures may be based on either concentration or MFI values. Although in our experiment, the two approaches yielded comparable misclassification rates and identified the same major cytokines for both the discrimination of bacterial stimuli and TLR ligands, small differences were evidenced in the implication of CXCL8. Notably, CXCL8 concentrations were the least well estimated due to a larger range of variation than defined by the assay manufacturer, leading to significant proportions of values outside the quantification range, whatever the stimulation condition. A high proportion of values outside the quantification range was also occasionally observed (e.g., for CCL4 and IFN-γ under HKSU stimulation conditions), but this did not interfere with the study results because only this condition was studied. Assigning the upper limit of quantification to values above the range of quantification led to an artificially reduced variance of CXCL8 values, which may explain its lower involvement in stimulus discrimination when concentration values were analyzed. In contrast, keeping MFI values guarantees the preservation of greater variability and better discrimination of the different stimuli based on CXCL8 levels (18). Nevertheless, saturating levels of CXCL8 were observed under the conditions of our experiments, meaning that the true differences between stimuli may not have been properly captured for this cytokine. Accurate assessment of CXCL-8 concentrations would have required using a different sample dilution for this target, which was logistically difficult. Another important piece of information is the identity of the cytokines that are discriminative among bacteria and among TLR ligands. A previous study by Urrutia et al. (43) showed that gene expression is mostly induced by four cytokines (e.g., IL-1β, TNF-α, IFN-γ, and IFN-β) that can capture the diversity of the response to various TLR ligands and microbes. Even if the multiplex assay excludes the quantification of IFN-β, it is worth noting that IL-1β, TNF-α, and IFN-γ were the most informative cytokines for distinguishing between bacteria. For TLR ligands, IL-1α and CXCL10, a type I interferon-induced protein, completed the list, reinforcing the view that this small set of cytokines plays a determining role in the initial response to microbes.

Finally, beyond the characterization of the stimulus-related cellular immune response, the development of a standardized whole blood assay and bead-based cytokine profiling also allows us to highlight the great inter-individual variability, within the same or between different stimulation conditions. In this respect, IFN-γ, TNF-α and IL-17A, IL-1β, and IL-1α were associated with the highest CV values (**Supplementary Table 4** **)** across all stimulation conditions that we tested, and may be of interest in defining different immune response profiles within our cow study sample. Investigating this inter-individual variability, however, requires further studies.

Assaying blood to interrogate the responsiveness of dairy cows to mammary pathogens may at first be surprising. However, milk cells respond poorly to stimulation by pathogens or their related MAMPS (59, 60), indicating that they are inappropriate samples for such investigation. For sure, blood composition is different from immune and non-immune (e.g., epithelial) cells located at the mammary epithelial barrier, but it contains a diversity of immune cells, and its composition is less influenced by previous or chronic infections responsible for mastitis. Despite some limitations, blood represents an immune tissue that is accessible and frequently used in other species, including humans, making comparisons to the same stimuli even possible and useful in comparative studies across species. This protocol may also apply to different conditions, like the response to vaccination, and may reveal a sound and effective approach for future bovine immunological studies.

## Data Availability Statement

The raw data supporting the conclusions of this article will be made available by the authors, without undue reservation.

## Ethics Statement

All procedures involving animals received approval from the Ethics Committee on Animal Experimentation (agreement No.2016082518447444), with all applicable provisions established by the European directive 2010/63/UE.

## Author Contributions

GF and PG acquired funding. GF designed the study. SB performed sample collection. RL, FL, and DB provided animal resource and information. SW and NC performed the laboratory analysis. JL and FC designed and performed the statistical analysis. JL, FC, and GF wrote the manuscript. All authors listed have made a substantial, direct, and intellectual contribution to the work and approved it for publication.

## Funding

The BOVIMMUNE research program was supported by APIS-GENE, who contributed financially to the development of the multiplex assay by MERCK-Millipore under the contract agreement with INRAE.

## Conflict of Interest

The authors declare that the research was conducted in the absence of any commercial or financial relationships that could be construed as a potential conflict of interest.

## Publisher’s Note

All claims expressed in this article are solely those of the authors and do not necessarily represent those of their affiliated organizations, or those of the publisher, the editors and the reviewers. Any product that may be evaluated in this article, or claim that may be made by its manufacturer, is not guaranteed or endorsed by the publisher.
