# Supplementary material for: Standardized Whole Blood Assay and Bead-Based Cytokine Profiling Reveal Commonalities and Diversity of the Response to Bacteria and TLR Ligands in Cattle
Source: Front Immunol. 2022 May 23;13:871780. doi: 10.3389/fimmu.2022.871780 (PMC9169910; doi:10.3389/fimmu.2022.871780)
Supplement: Supplementary file 1 [file DataSheet_1.pdf]

Supplementary Material

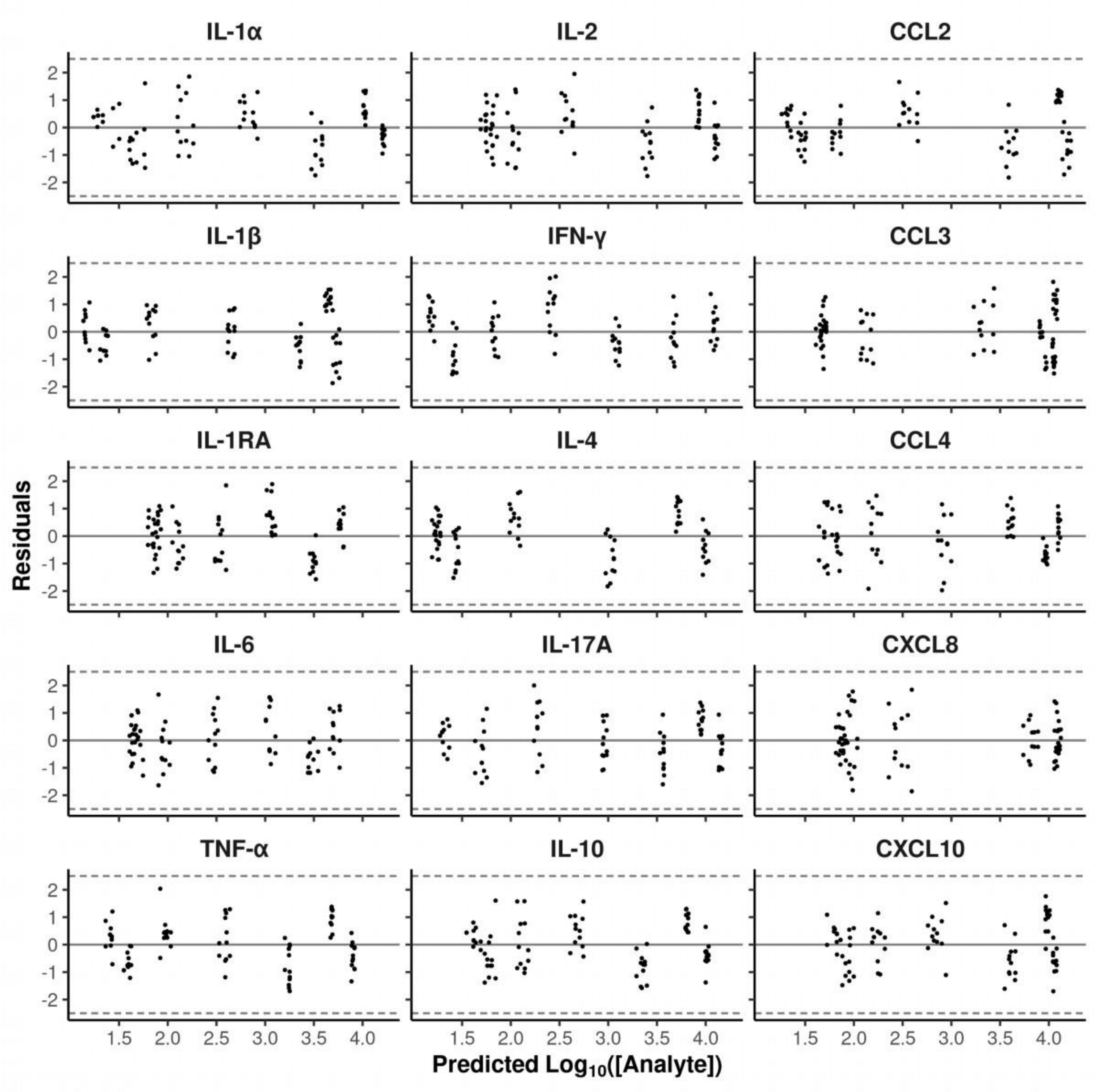

Supplementary Figure 1. Distribution of standardized residuals for each cytokine. The x and y-axis correspond to the predicted cytokine concentrations in Log10 scale and the residuals (difference between the predicted and the observed cytokine concentrations), respectively. Each point represents a standard sample. The solid line corresponds to a perfect match between observed and expected concentrations and the dashed lines correspond to the upper and lower limits of acceptation.

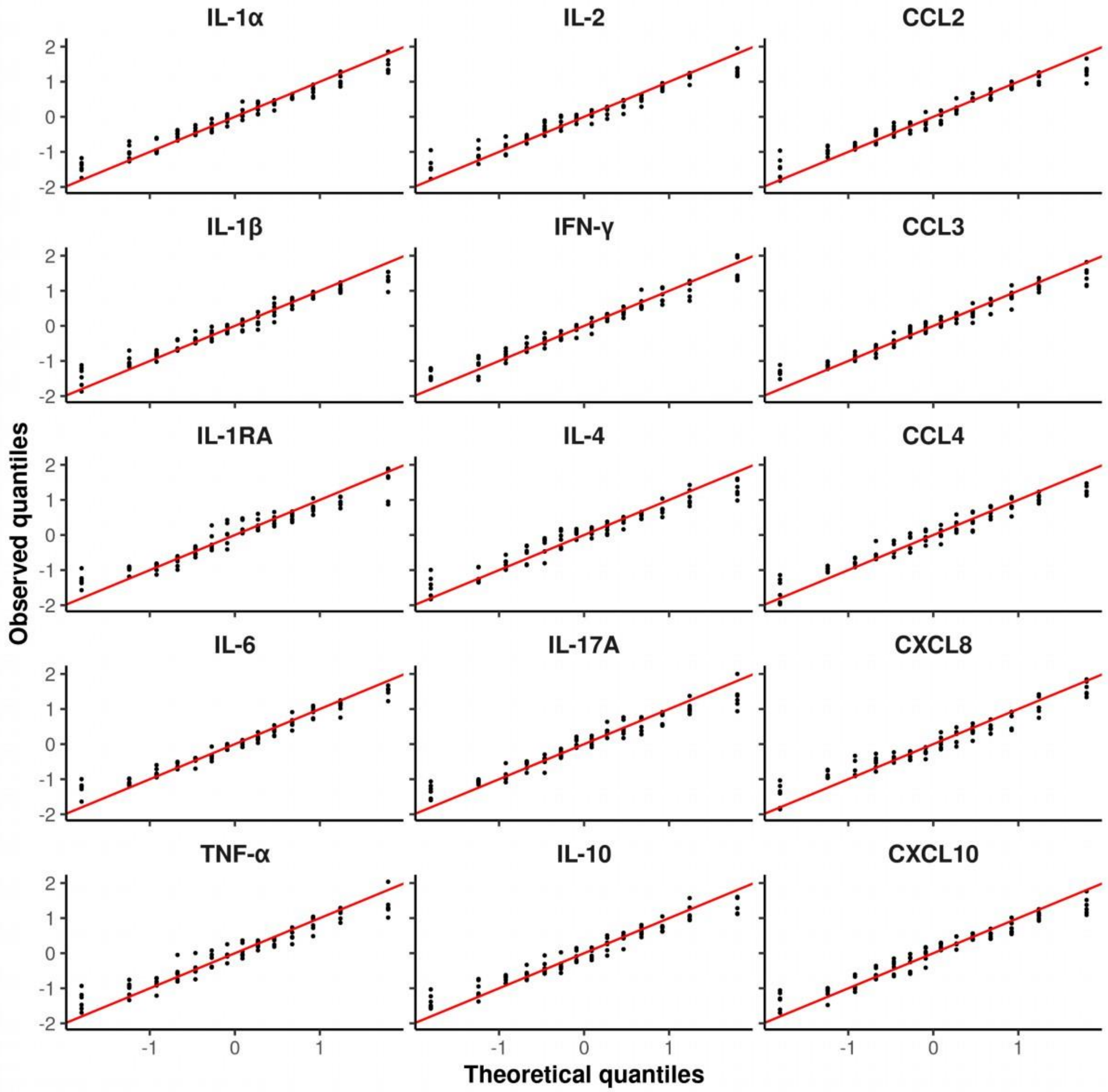

Supplementary Figure 2. Quantile-quantile diagrams of standardized residuals for each analyte. The x and y-axis represent the quantiles of a standard normal distribution and those of standardized residuals distributions, respectively. If standardized residuals follow a normal distribution, the dots are supposed to be distributed close to the red line.

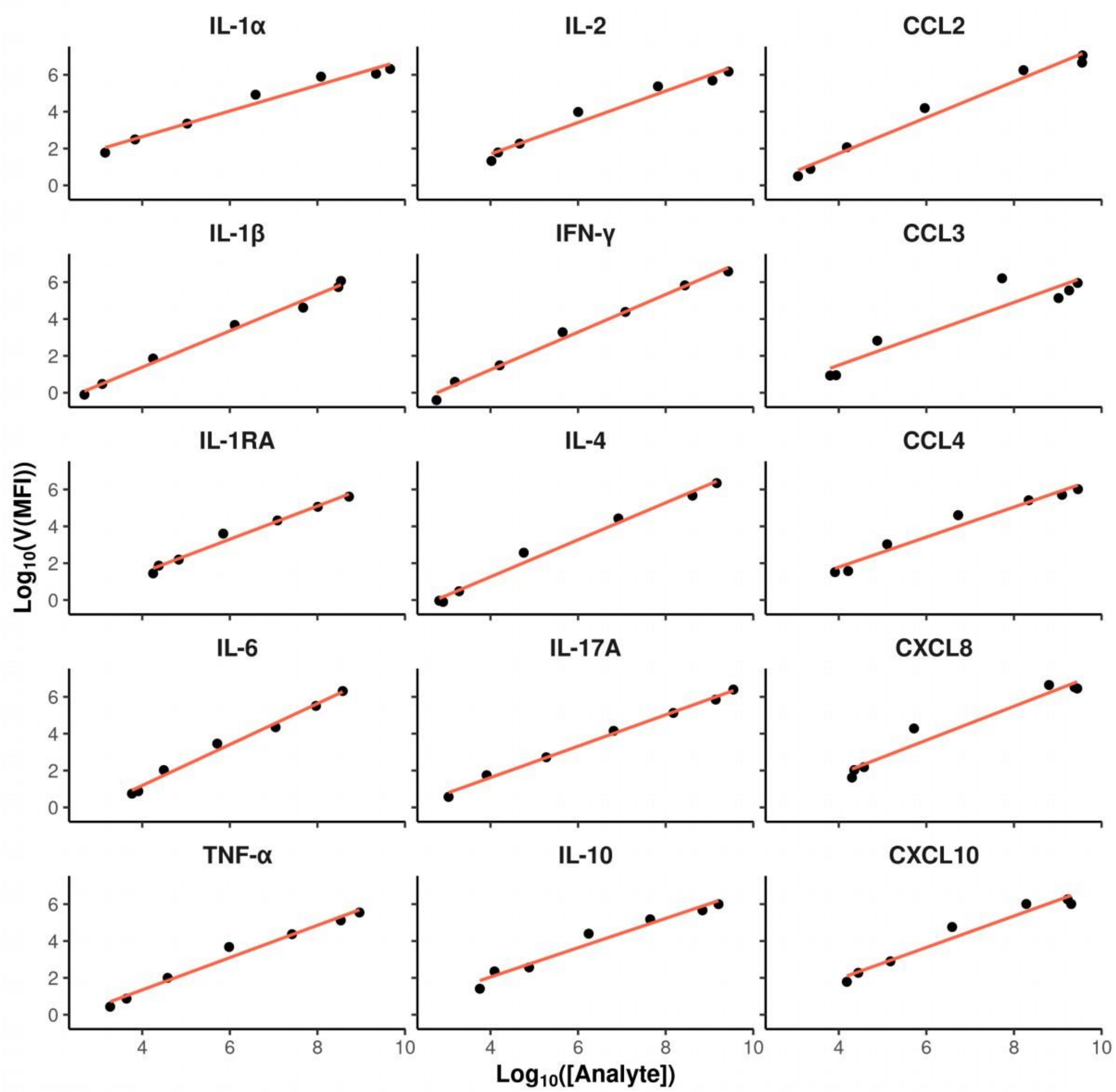

Supplementary Figure 3. Power-of-the-mean (POM) relationship between the variance and the mean MFI values. The x and y-axis represent the MFI values and their variances, on the Log10 scale, respectively. Each point corresponds to a standard and the red lines represent the linear regression between the MFI variance and the average MFI values of the standards, on the Log10 scale.

Supplementary Table 1. Coefficients of the logistic functions used for fitting the dose-response curves. A 5-parameter logistic function was implemented except when is indicated 'NA' showing that a 4-parameter logistic function was used.

Analyte	Plate	b	c	d	e	f
**IL-1α**	1	-0.69	1.24	4.31	2.22	0.63
**IL-1α**	3	-0.86	0.67	4.27	2.6	0.31
**IL-1α**	5	-0.86	0.66	4.29	2.67	0.31
**IL-1α**	7	-1.27	-0.02	4.23	3.04	0.13
**IL-1α**	9	-0.96	0.42	4.29	2.84	0.22
**IL-1α**	11	-0.73	0.89	4.33	2.48	0.41
**IL-2**	1	-0.88	1.72	4.15	3.54	0.9
**IL-2**	3	-0.91	1.71	4.14	3.55	0.87
**IL-2**	5	-0.96	1.72	4.15	3.68	0.77
**IL-2**	7	-0.9	1.67	4.16	3.63	0.87
**IL-2**	9	-0.94	1.71	4.17	3.69	0.79
**IL-2**	11	-0.79	1.77	4.21	3.4	1.15
**CCL2**	1	-1.55	1.14	4.15	3.32	0.28
**CCL2**	3	-1.33	1.25	4.17	3.12	0.42
**CCL2**	5	-1.31	1.23	4.21	3.22	0.43
**CCL2**	7	-1.52	1.09	4.2	3.39	0.29
**CCL2**	9	-1.48	1.17	4.22	3.31	0.33
**CCL2**	11	-1.09	1.26	4.25	3.04	0.55
**IL-1β**	1	-0.84	1.13	3.73	2.43	0.96
**IL-1β**	3	-0.99	0.99	3.71	2.74	0.55
**IL-1β**	5	-0.94	1.03	3.75	2.69	0.67
**IL-1β**	7	-0.82	1.06	3.77	2.46	0.98
**IL-1β**	9	-0.92	1.03	3.79	2.71	0.67
**IL-1β**	11	-0.82	1.04	3.81	2.52	0.84
**IFN-γ**	1	-0.33	1.16	4.82	-1.42	8.04
**IFN-γ**	3	-0.49	0.97	4.45	1.49	NA
**IFN-γ**	5	-0.47	0.94	4.57	1.57	NA
**IFN-γ**	7	-0.44	0.88	4.65	1.61	NA
**IFN-γ**	9	-0.45	0.9	4.65	1.59	NA
**IFN-γ**	11	-0.43	0.86	4.68	1.53	NA
**CCL3**	1	-1.07	1.66	4.07	2.39	2.92
**CCL3**	3	-1.41	1.64	4.05	2.88	NA
**CCL3**	5	-1.04	1.68	4.08	2.13	5.37
**CCL3**	7	-1.17	1.6	4.08	2.85	1.29
**CCL3**	9	-1.17	1.69	4.09	2.63	2.09
**CCL3**	11	-1.46	1.65	4.05	2.89	NA
**IL-1RA**	1	-0.65	1.75	3.92	3.3	NA
**IL-1RA**	3	-0.4	1.85	4.14	0.48	11.72
**IL-1RA**	5	-0.44	1.85	4.11	1.47	5.78
**IL-1RA**	7	-0.42	1.79	4.13	1.18	6.95
**IL-1RA**	9	-0.41	1.86	4.19	1.17	7
**IL-1RA**	11	-0.38	1.87	4.21	-0.53	23.86
**IL-4**	1	-0.78	1.24	4.09	3.03	1.51
**IL-4**	3	-0.75	1.25	4.09	2.8	2.02
**IL-4**	5	-0.79	1.22	4.12	3.05	1.52

Analyte	Plate	b	c	d	e	f
**IL-4**	7	-0.85	1.18	4.08	3.26	1.16
**IL-4**	9	-0.86	1.22	4.11	3.27	1.16
**IL-4**	11	-0.76	1.2	4.16	3.07	1.41
**CCL4**	1	-0.66	1.65	4.18	2.28	1.44
**CCL4**	3	-0.71	1.69	4.16	2.32	1.37
**CCL4**	5	-0.81	1.64	4.15	2.62	0.97
**CCL4**	7	-0.79	1.58	4.15	2.64	0.96
**CCL4**	9	-0.83	1.65	4.14	2.61	0.98
**CCL4**	11	-0.66	1.7	4.2	2.11	1.68
**IL-6**	1	-0.45	1.62	3.96	-0.45	12.24
**IL-6**	3	-0.48	1.63	3.88	0.23	6.61
**IL-6**	5	-0.51	1.63	3.91	0.89	3.75
**IL-6**	7	-0.49	1.6	3.95	0.78	4.24
**IL-6**	9	-0.45	1.65	4.06	0.44	5.37
**IL-6**	11	-0.64	1.56	3.91	2.16	NA
**IL-17A**	1	-0.54	1.02	4.29	1.35	0.93
**IL-17A**	3	-0.67	0.52	4.24	2.04	0.39
**IL-17A**	5	-0.63	0.59	4.28	1.93	0.47
**IL-17A**	7	-0.44	1.06	4.41	0.44	2.14
**IL-17A**	9	-0.62	0.54	4.3	1.96	0.45
**IL-17A**	11	-0.53	0.75	4.35	1.63	0.67
**CXCL8**	1	-3.14	1.86	4.06	2.8	0.29
**CXCL8**	3	-2.4	1.88	4.08	2.6	0.51
**CXCL8**	5	-2.67	1.84	4.1	2.76	0.4
**CXCL8**	7	-7.26	1.8	4.08	2.98	0.11
**CXCL8**	9	-2.77	1.89	4.1	2.79	0.38
**CXCL8**	11	-2.11	1.91	4.12	2.64	0.52
**TNF-α**	1	-0.68	1.27	4	3.46	0.8
**TNF-α**	3	-0.66	1.28	4.01	3.43	0.82
**TNF-α**	5	-0.59	1.36	4.06	3.03	1.36
**TNF-α**	7	-0.67	1.21	4.03	3.55	0.77
**TNF-α**	9	-0.6	1.3	4.08	3.24	1.06
**TNF-α**	11	-0.54	1.3	4.11	3.05	1.23
**IL-10**	1	-0.74	1.51	4.1	3.03	0.69
**IL-10**	3	-0.76	1.48	4.08	2.98	0.68
**IL-10**	5	-0.71	1.52	4.13	2.93	0.83
**IL-10**	7	-0.85	1.32	4.09	3.37	0.45
**IL-10**	9	-0.76	1.49	4.13	3.11	0.65
**IL-10**	11	-0.64	1.59	4.17	2.8	0.94
**CXCL10**	1	-1.18	1.73	4.06	2.72	0.45
**CXCL10**	3	-1.2	1.73	4.06	2.67	0.45
**CXCL10**	5	-1.11	1.72	4.08	2.68	0.51
**CXCL10**	7	-1.36	1.56	4.05	2.93	0.31
**CXCL10**	9	-1.43	1.66	4.08	2.94	0.3
**CXCL10**	11	-1.03	1.77	4.1	2.59	0.55

Supplementary Table 2. Proportions of individuals outside the range of quantification.
They are given in percentage for each stimulus and cytokine. The columns "lower" and "upper" give respectively the proportions of individuals below and above the ranges of quantification.

	CTL (%)		HKEC (%)		HKSA (%)		HKSU (%)		FSL-1 (%)		GDQ (%)		LPS (%)	
**Analyte**	**lower**	**upper**	**lower**	**upper**	**lower**	**upper**	**lower**	**upper**	**lower**	**upper**	**lower**	**upper**	**lower**	**upper**
**CCL2**	0	4.7	0	12.1	0	8.4	0	0	0	34.6	0	2.8	0	22.4
**CCL3**	0	0	0	0	0	0	0	0	0	0	0	0.9	0	0
**CCL4**	0	0	0	0.9	0	2.8	0	23.4	0	1.9	0	14	0	2.8
**CXCL8**	0	3.7	0	92.5	0	54.2	0	45.8	0	76.6	0	33.6	0	86
**CXCL10**	0	0	0	0	0	0	0	0	0	0	0	11.2	0	0
**IFN-γ**	0.9	0	0	0	0	0.9	0	32.7	0	0.9	0	4.7	0	5.6
**IL-10**	1.9	0	0	0	0	0	0	0	0.9	0	0	0	0	0
**IL-17A**	43.9	0	0	0	0.9	0	0	0	3.7	0	3.7	0	1.9	0
**IL-1RA**	7.5	0	0	0	0	0	0	0	0	0	0	0	0	0
**IL-1α**	28	0	0	0	0.9	0	0	0	2.8	0	0.9	0	1.9	0
**IL-1β**	21.5	0	0	0	0	0	0	0	0	0	0	0	0	0
**IL-2**	8.4	0	0	0	1.9	0	1.9	0	0.9	0	1.9	0	1.9	0
**IL-4**	0	0	0	0	0	0	0	0	0	0	0	0	0	0
**IL-6**	2.8	0	0	0	0.9	0	0	0	0.9	0	0	0	0.9	0
**TNF-α**	0	0	0	0	0	0	0	0	0	0	0	0	0	0

Supplementary Table 3. Analyte concentrations in the null condition. Quartile values are shown for each analyte (in pg/mL).

Analyte	1st quartile	Median	3rd quartile
**CCL2**	1,311	2,479	5,332
**CCL3**	226	373	722
**CCL4**	446	1,133	2,048
**CXCL8**	69	98	153
**CXCL10**	98	119	154
**IFN-γ**	0	0	1
**IL-10**	18	28	44
**IL-17A**	0	0	1
**IL-1RA**	65	128	200
**IL-1α**	1	1	2
**IL-1β**	5	7	10
**IL-2**	73	113	165
**IL-4**	97	121	165
**IL-6**	8	9	12
**TNF-α**	42	70	123

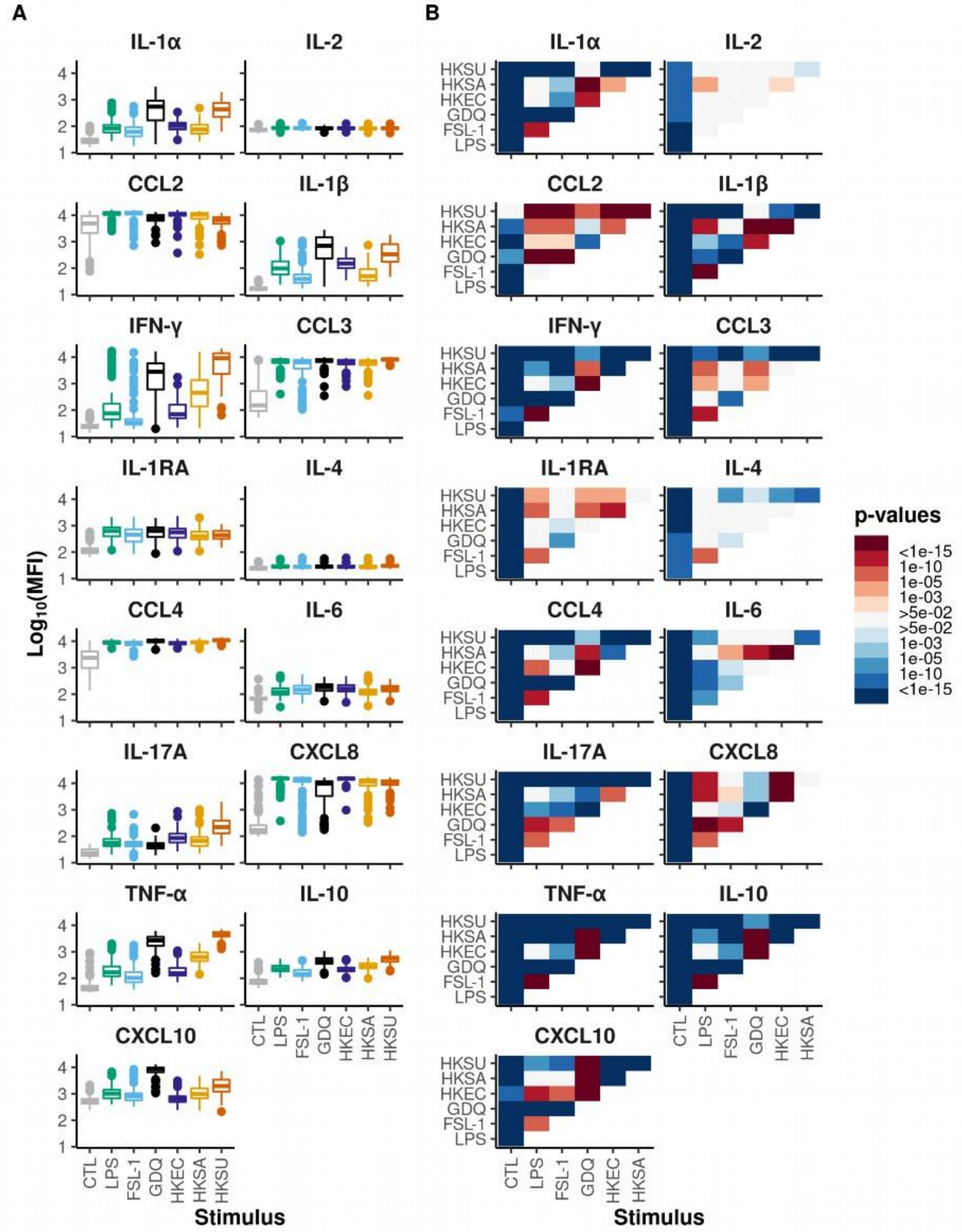

Supplementary Figure 4. (A) Distributions of raw Log10-MFI values for each cytokine under different stimulus conditions, obtained from 107 cows. **(B)** Inter-stimuli pairwise comparisons for each cytokine, using the Wilcoxon signed-rank test with Holm-Bonferroni correction for multiple comparisons. A gradient of blue (conversely red) color indicates that the Log10-MFI values under the condition on the horizontal x-axis are lower (conversely greater) than the ones under the condition on the vertical y-axis.

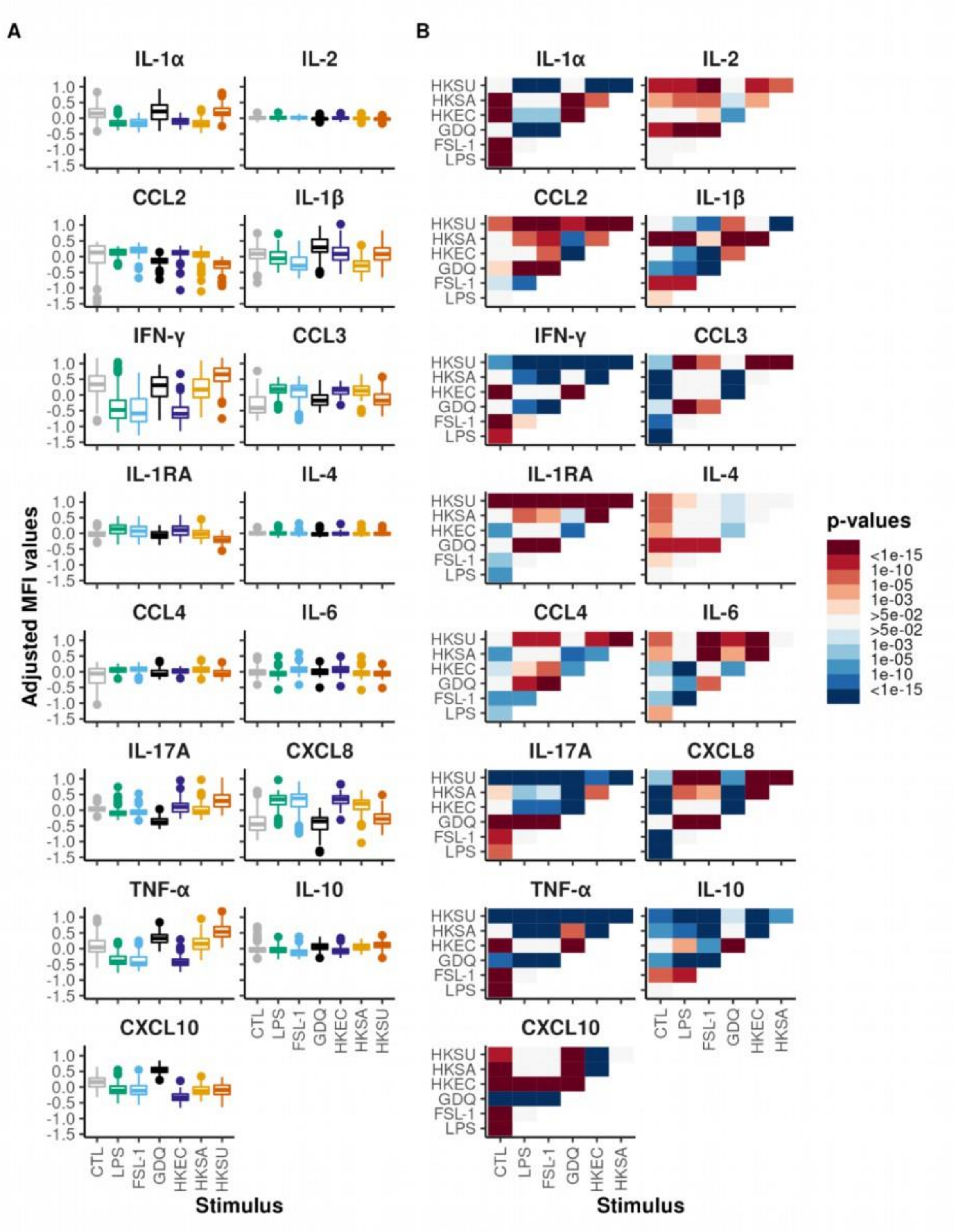

Supplementary Figure 5. (A) Distributions of adjusted to the mean MFI values for each cytokine under different stimulus conditions, obtained from 107 cows. **(B)** Inter-stimuli pairwise comparisons for each cytokine, using the Wilcoxon signed-rank test with Holm-Bonferroni correction for multiple comparisons. A gradient of blue (conversely red) color indicates that the adjusted MFI values under the condition on the horizontal x-axis are lower (conversely greater) than the ones under the condition on the vertical y-axis.

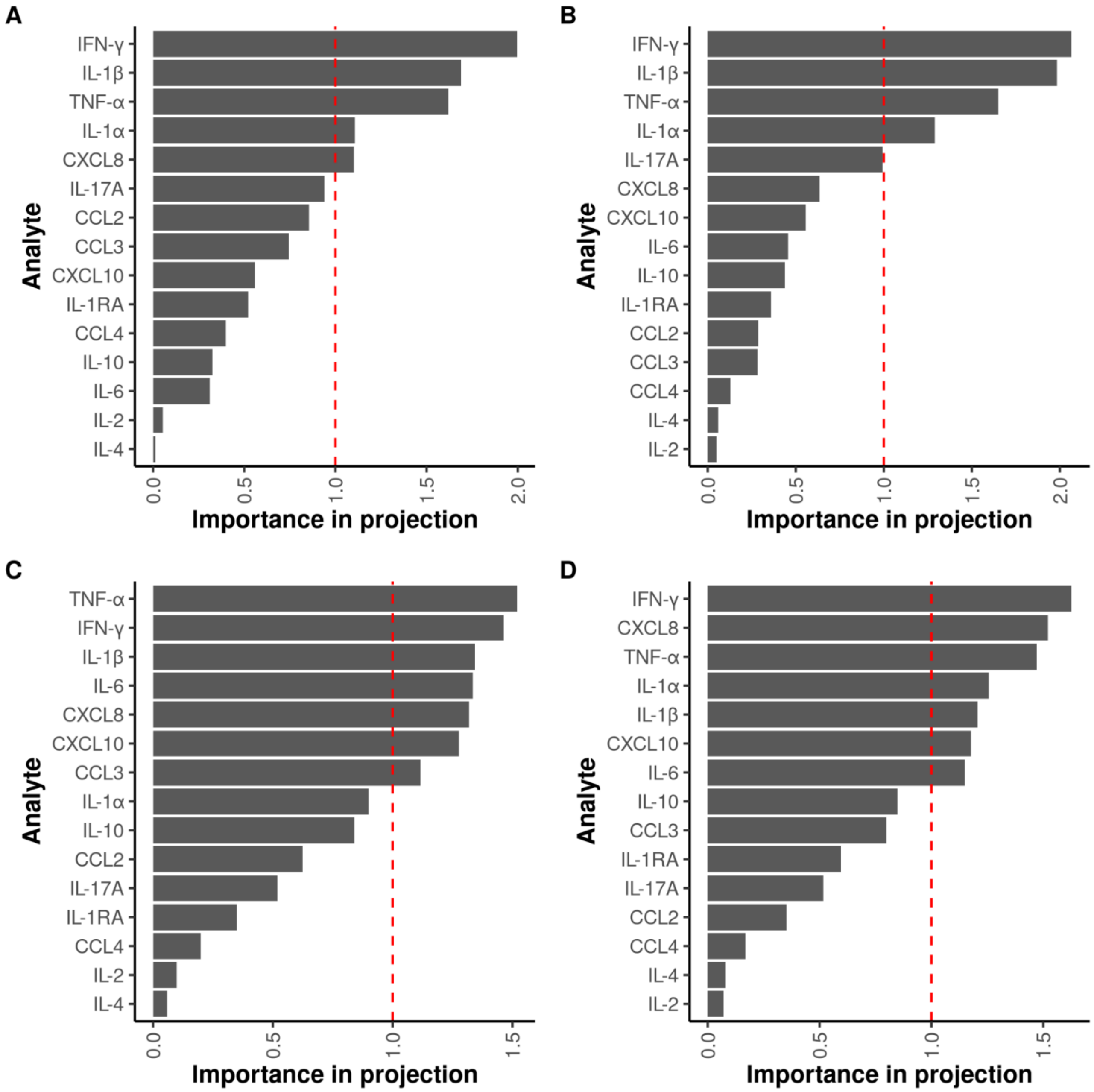

Supplementary Figure 6. Horizontal barplot of the variable importance in projection (VIP) scores obtained for the multilevel PLS-DA realised on bacterial adjusted (**A**) and unadjusted MFI values (**B**), and TLR ligands adjusted (**C**) and unadjusted MFI values (**D**). VIP scores reflect the relative importance of each variable to discriminate the various stimulation conditions. A variable with a VIP score above 1 (vertical red dash line) plays a major role in the prediction model.

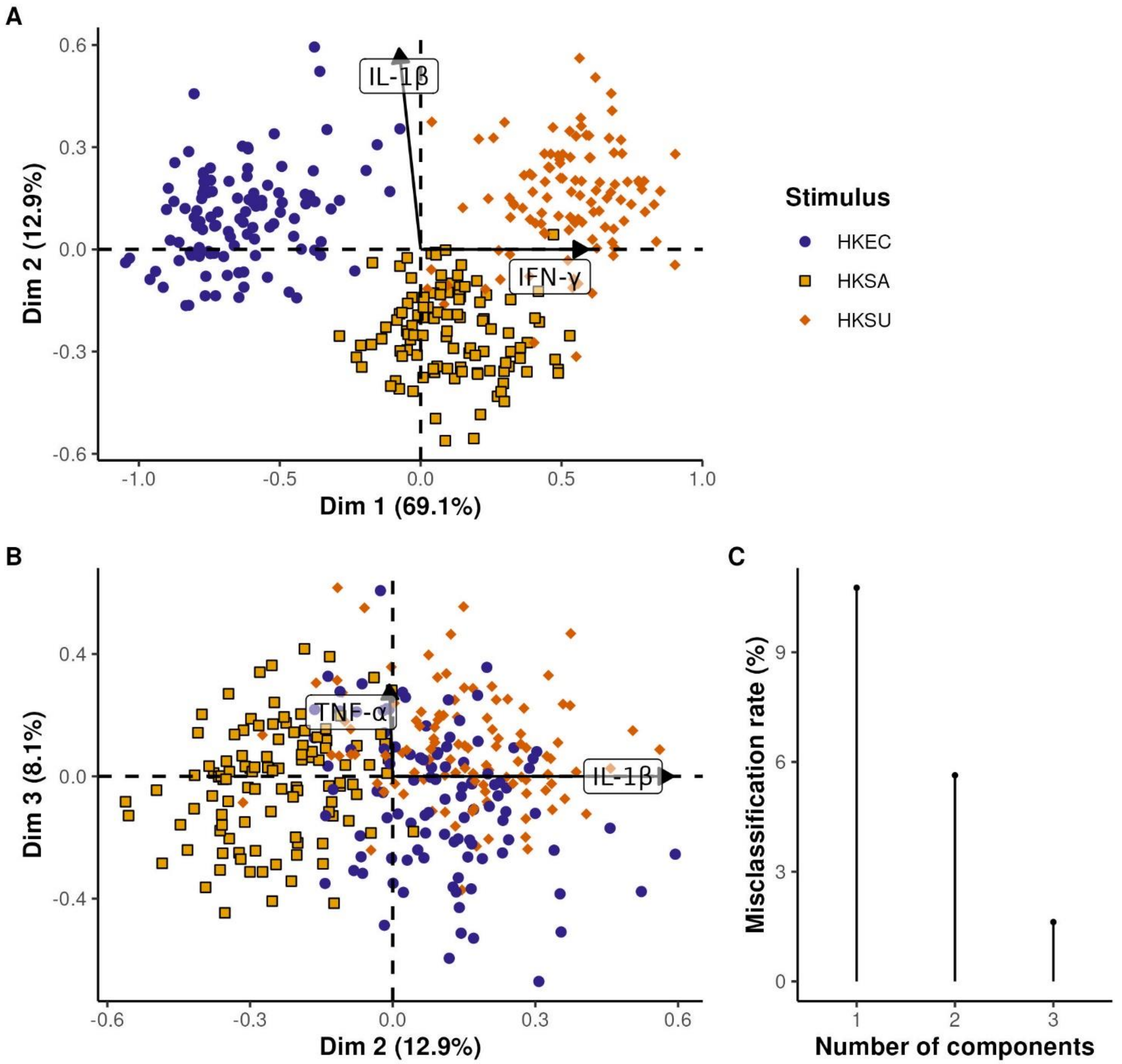

Supplementary Figure 7. Multilevel sparse PLS-DA on bacterial stimuli adjusted MFI values reveals specific patterns of expression according to the bacteria species. On the plot of the individuals (A, B), each dot represents an individual cow and the corresponding stimulus is defined by the different colors and shapes. The x and y-axis correspond to the sparse PLS-DA components. The proportion of variance explained by each principal component is given in brackets. (C) Misclassification rate (MR) achieved given the number of sparse PLS-DA components: only 3 cytokines are sufficient to reach an almost perfect classification rate with 2 (MR = 5.63%) or 3 components (MR = 1.62 %).

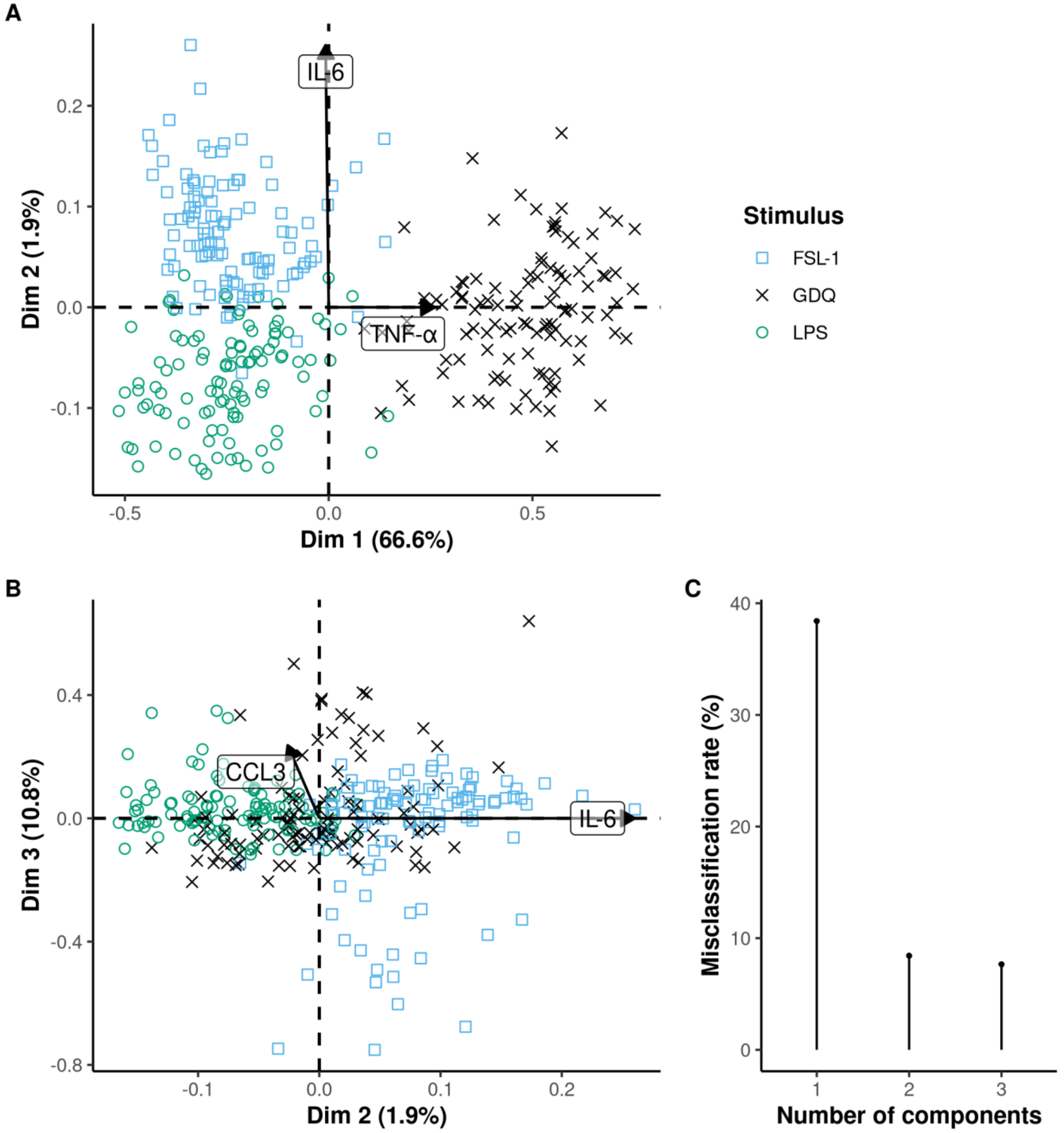

Supplementary Figure 8. Multilevel sparse PLS-DA on TLR ligands adjusted MFI values identifies commonalities between TLR2 and TLR4 engagements. On the plots of the individuals (A, B), each dot represents an individual cow and the corresponding stimulus is defined by the different colors and shapes. The x and y-axis correspond to the sparse PLS-DA components. Misclassification rate (MR) achieved given the number of sparse PLS-DA components: only 3 cytokines are sufficient to reach a classification error rate lower than 10% (MR = 8.4 %) on the first two components.

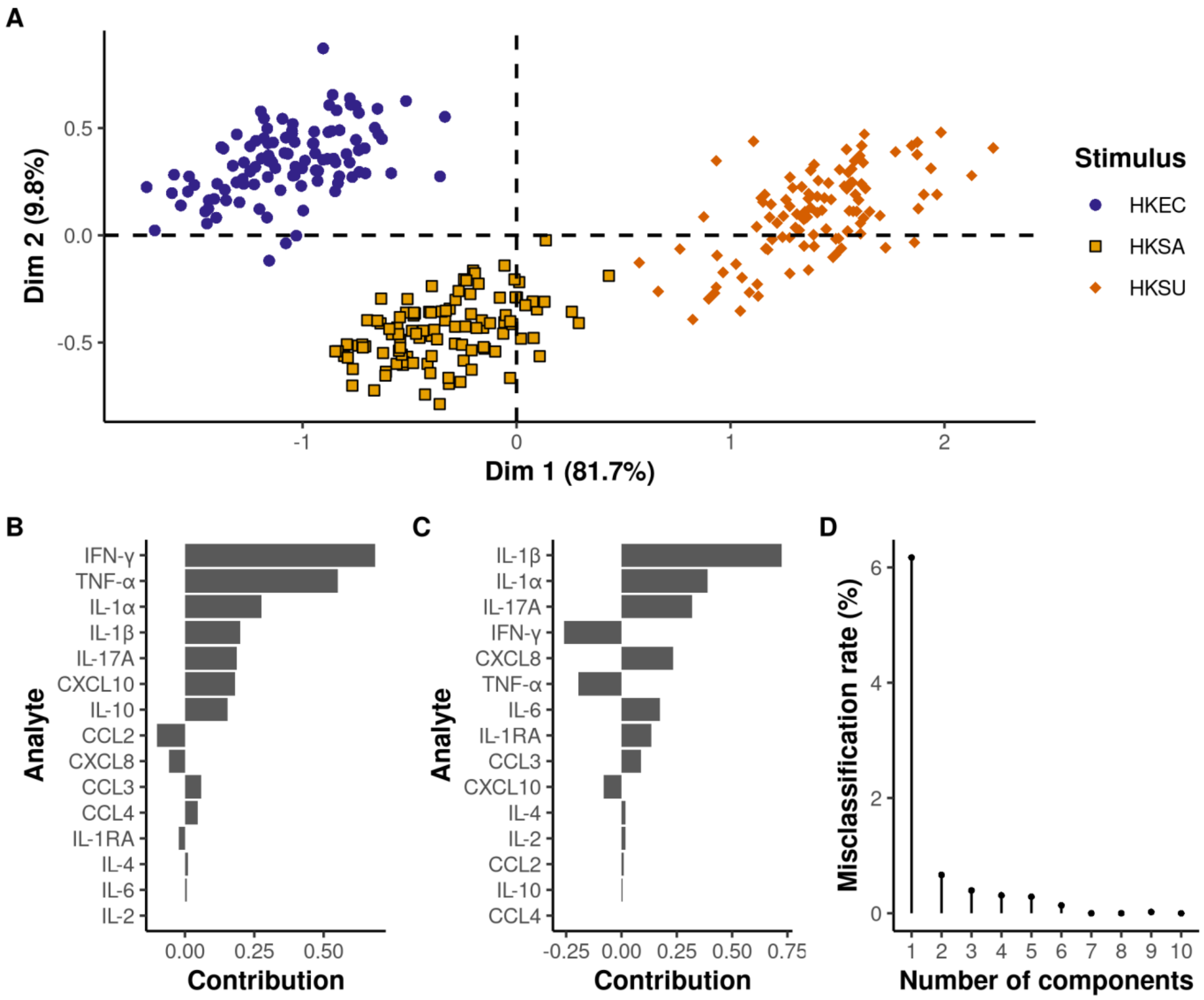

Supplementary Figure 9. Multilevel PLS-DA on bacterial stimuli unadjusted MFI values reveals specific expression patterns depending on the bacteria species. On the plot of the individuals (**A**), each dot represents an individual cow and the corresponding stimulus is defined by various colors and shapes. The x and y-axis correspond to the first and second PLS-DA components, respectively. The proportion of variance explained by each principal component is written in brackets. The contribution of the cytokines to the first (**B**) and second (**C**) principal components are represented by a horizontal barplot. The two first principal components are sufficient to reach an almost perfect misclassification rate (**D**).

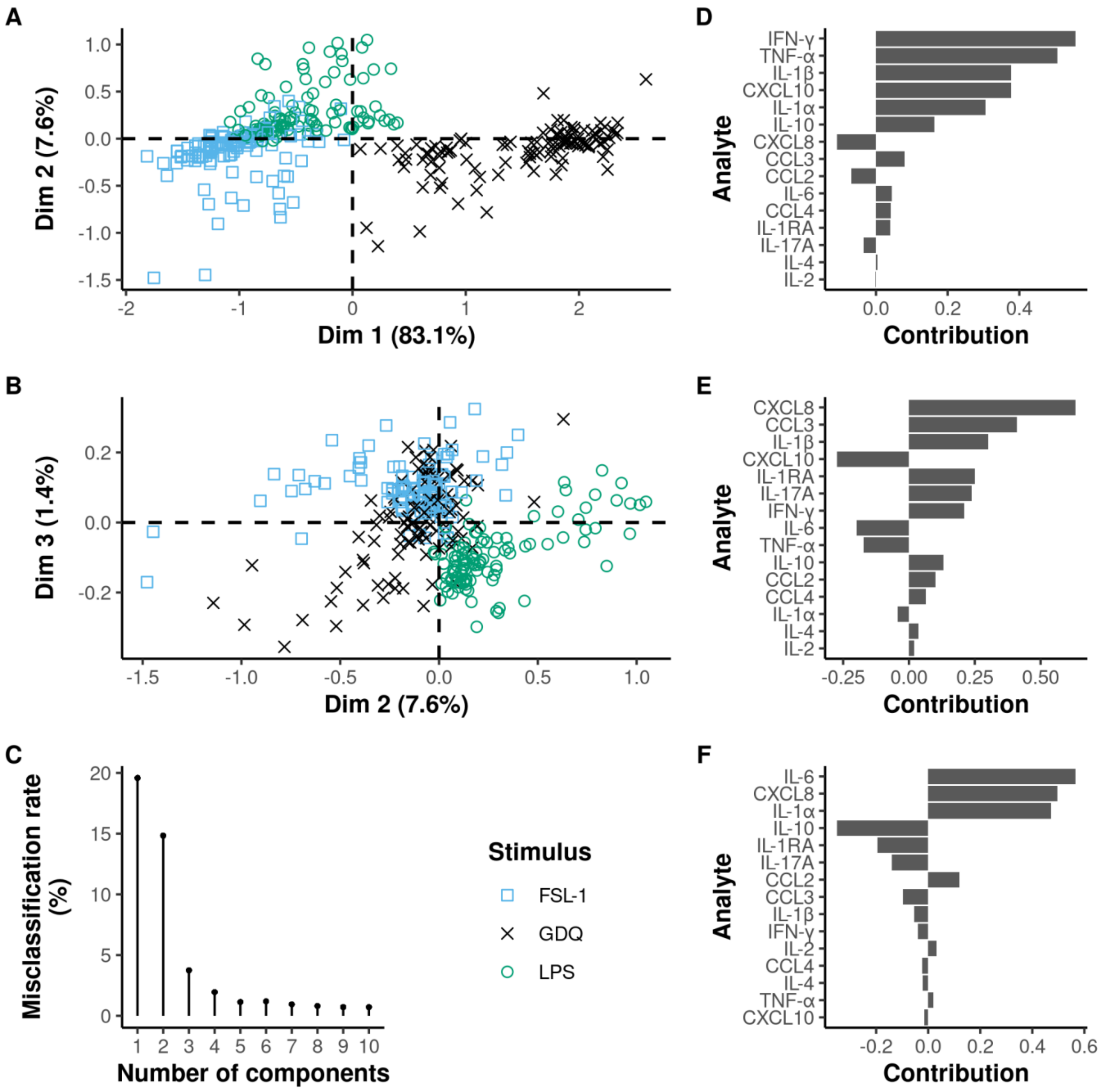

Supplementary Figure 10. Multilevel PLS-DA on TLR ligands unadjusted MFI values identifies commonalities between TLR2/6 and TLR4 engagements. On the plots of the individuals (**A**, **B**), each dot represents an individual cow and the corresponding stimulus is defined by the different colors and shapes. The x and y-axis correspond to the PLS-DA components, respectively. The proportion of variance explained by each principal component is written in brackets. The contribution of the cytokines to the first (**D**), second (**E**) and third (**F**) principal components are represented by a horizontal barplot. The three first principal components are sufficient to reach a misclassification rate below 10% (**C**).

Supplementary Table 4. Raw MFI values coefficient of variation (CV) in percentage for each cytokine and stimulation condition. CTL, FSL-1, GDQ, HKEC, HKSA, HKSU and LPS stand for control, fibroblast-stimulating lipopeptide 1, gardiquimod, heat-killed *Escherichia coli*, heat-killed *Staphylococcus aureus*, heat-killed *Streptococcus uberis* and lipopolysaccharide stimuli, respectively.

		**Stimulus**						
	**Analyte**	**CTL**	**FSL-1**	**GDQ**	**HKEC**	**HKSA**	**HKSU**	**LPS**
**CV (%)**	**CCL2**	74	27	34	27	37	44	25
	**CCL3**	205	54	26	30	33	15	28
	**CCL4**	83	25	15	17	16	13	16
	**CXCL8**	280	46	60	17	42	34	25
	**CXCL10**	47	114	33	55	69	65	92
	**IFN-γ**	38	384	99	155	176	73	258
	**IL-10**	64	47	37	35	33	33	38
	**IL-17A**	38	109	49	94	128	94	119
	**IL-1RA**	65	76	55	62	63	47	59
	**IL-1α**	45	98	94	46	71	77	102
	**IL-1β**	19	179	86	64	114	87	118
	**IL-2**	16	15	13	15	15	13	14
	**IL-4**	19	19	17	18	19	16	18
	**IL-6**	45	52	40	43	50	35	47
	**TNF-α**	172	142	51	76	59	29	125
